# Supplementary material for: Outdoor Residual Insecticide Spraying (ODRS), a New Approach for the Control of the Exophilic Vectors of Human Visceral Leishmaniasis: Phlebotomus orientalis in East Africa
Source: PLoS Negl Trop Dis. 2020 Oct 20;14(10):e0008774. doi: 10.1371/journal.pntd.0008774 (PMC7598920; doi:10.1371/journal.pntd.0008774)
Supplement: S1 Table — (DOCX) [file pntd.0008774.s001.docx]

**S1 Table:** Absolute numbers of *Phlebotomus orientalis* sandflies captured per trap night before and after outdoor residual insecticide spraying of exterior walls of sleeping huts, and household boundary fences (ODRS), or boundary fences alone (RODRS), in Gedarif state, eastern Sudan. 9a) Experiment 1 conducted in June 2016 in Jebel-Algana village; (b) Experiment 2 conducted in May-June 2007 in Umsalala village; (c) Experiment 3 conducted in March-June 2017 in Jebel-Algana village. For Experiment 2 and 3, numbers were calculated over 43 and 76 days follow-up, respectively.

1. **Experiment 1:**

| **Trap site** | **Trap type^1^** | **Control (non-intervention) houses** | | | | **ODRS Treated houses** | | | |
| --- | --- | --- | --- | --- | --- | --- | --- | --- | --- |
|  |  | Total *P. orientalis* | Male *P. orientalis* | Female *P. orientalis* | n traps nights | Total *P. orientalis* | Male *P. orientalis* | Female *P. orientalis* | n traps nights |
| **pre-intervention** |  |  |  |  |  |  |  |  |  |
|  |  |  |  |  |  |  |  |  |  |
| Inside houses | KD | 4 | 0 | 4 | 8 | 7 | 1 | 6 | 8 |
| Outdoor | LT | 17 | 6 | 11 | 32 | 42 | 11 | 31 | 32 |
| Outdoor | ST | 216 | 77 | 139 | 32 | 388 | 172 | 216 | 32 |
| Peridomestic | ST | 195 | 66 | 129 | 32 | 341 | 138 | 203 | 32 |
| sum |  | 432 | 149 | 283 | 104 | 778 | 322 | 456 | 104 |
|  |  |  |  |  |  |  |  |  |  |
| **post-intervention** |  |  |  |  |  |  |  |  |  |
| Experiment 1 |  |  |  |  |  |  |  |  |  |
| Inside houses | KD | 13 | 3 | 10 | 8 | 2 | 0 | 2 | 8 |
| Outdoor | LT | 7 | 1 | 6 | 32 | 0 | 0 | 0 | 32 |
| Outdoor | ST | 113 | 41 | 72 | 32 | 29 | 7 | 22 | 32 |
| Peridomestic | ST | 104 | 31 | 73 | 32 | 22 | 4 | 18 | 32 |
| sum |  | 237 | 76 | 161 | 104 | 53 | 11 | 42 | 104 |

1. Trap types: KD= Insecticide knockdown collection of flies found resting in the rooms; LT = CDC light traps operated between 18:00-6:00 am;

10 sticky-paper traps placed horizontally on the ground at 0.5m from each other.

**(b) Experiment-2**

|  |  |  |  |  |  |  |  |  |  |
| --- | --- | --- | --- | --- | --- | --- | --- | --- | --- |
| **Trap site** | **Trap type^1^** | **Control (non-intervention) houses** | | | | **ODRS Treated houses** | | | |
|  |  | Total *P. orientalis* | Male *P. orientalis* | Female *P. orientalis* | n traps nights | Total *P. orientalis* | Male *P. orientalis* | Female *P. orientalis* | n traps nights |
| **pre-intervention** |  |  |  |  |  |  |  |  |  |
| Outdoor | LT | 304 | 234 | 70 | 16 | 353 | 271 | 82 | 16 |
| Outdoor | ST | 206 | 152 | 54 | 14 | 280 | 210 | 70 | 14 |
| Peridomestic | ST | 254 | 186 | 68 | 14 | 279 | 209 | 70 | 14 |
| sum |  | 764 | 572 | 192 | 44 | 912 | 690 | 222 | 44 |
| **post-intervention** |  |  |  |  |  |  |  |  |  |
| Outdoor | LT | 1930 | 1473 | 457 | 103 | 25 | 14 | 11 | 103 |
| Outdoor | ST | 1537 | 1225 | 312 | 76 | 81 | 66 | 15 | 76 |
| Peridomestic | ST | 1591 | 1284 | 307 | 76 | 101 | 72 | 29 | 76 |
| sum |  | 5058 | 3982 | 1076 | 255 | 207 | 152 | 55 | 255 |

1. Trap types: KD= Insecticide knockdown collection of flies found resting in the rooms; LT = CDC light traps operated between 18:00-6:00 am;

10 sticky-paper traps placed horizontally on the ground at 0.5m from each other.

**(C )** Experiment-3):

| **Trap site** | **Trap type^1^** | **Control (non-intervention) houses** | | | | **ODRS treated houses** | | | | **RODRS treated houses** | | | |
| --- | --- | --- | --- | --- | --- | --- | --- | --- | --- | --- | --- | --- | --- |
|  |  | **Total *P. orientalis*** | **Male *P. orientalis*** | **Female *P. orientalis*** | **n traps nights** | **Total *P. orientalis*** | **Male *P. orientalis*** | **Female *P. orientalis*** | **n traps nights** | **Total *P. orientalis*** | **Male *P. orientalis*** | **Female *P. orientalis*** | **n traps nights** |
| **pre-intervention** |  |  |  |  |  |  |  |  |  |  |  |  |  |
| Outdoor | LT | 159 | 106 | 53 | 32 | 144 | 79 | 65 | 32 | 158 | 94 | 64 | 32 |
| Outdoor | ST | 109 | 54 | 55 | 32 | 189 | 95 | 94 | 32 | 165 | 86 | 79 | 32 |
| Peridomestic | ST | 111 | 46 | 65 | 32 | 155 | 80 | 75 | 32 | 132 | 59 | 73 | 32 |
| sum |  | 379 | 206 | 173 | 96 | 488 | 254 | 234 | 96 | 455 | 239 | 216 | 96 |
| **post-intervention** |  |  |  |  |  |  |  |  |  |  |  |  |  |
| Outdoor | LT | 119 | 65 | 54 | 83 | 57 | 34 | 23 | 85 | 80 | 63 | 17 | 85 |
| Outdoor | ST | 175 | 98 | 77 | 80 | 67 | 38 | 29 | 79 | 157 | 84 | 73 | 80 |
| Peridomestic | ST | 210 | 113 | 97 | 80 | 60 | 35 | 25 | 79 | 125 | 66 | 59 | 80 |
| sum |  | 504 | 276 | 228 | 243 | 184 | 107 | 77 | 243 | 362 | 213 | 149 | 245 |

1. Trap types: KD= Insecticide knockdown collection of flies found resting in the rooms; LT = CDC light traps operated between 18:00-6:00 am;

10 sticky-paper traps placed horizontally on the ground at 0.5m from each other.
