# Supplementary material for: Outdoor Residual Insecticide Spraying (ODRS), a New Approach for the Control of the Exophilic Vectors of Human Visceral Leishmaniasis: Phlebotomus orientalis in East Africa
Source: PLoS Negl Trop Dis. 2020 Oct 20;14(10):e0008774. doi: 10.1371/journal.pntd.0008774 (PMC7598920; doi:10.1371/journal.pntd.0008774)
Supplement: S2 Table — (a) Experiment 1 conducted in June 2016 in Jebel-Algana village; (b) Experiment 2 conducted in May-June 2017 in Umsalala village; (c) Experiment-3 conducted in March-June 2017 in Jebel-Algana village. For Experiments 2 and 3, median values were calculated over 43- and 76-days follow-up respectively. (DOCX) [file pntd.0008774.s002.docx]

**S2 Table:** Median (IQR) numbers of *Phlebotomus orientalis* sandflies captured per trap night before and after outdoor residual insecticide spraying of exterior walls of sleeping huts, and household boundary fences (ODRS), or boundary fences alone (RODRS), in Gedarif state, eastern Sudan. (a) Experiment 1 conducted in June 2016 in Jebel-Algana village; (b) Experiment 2 conducted in May-June 2017 in Umsalala village; (c) Experiment-3 conducted in March-June 2017 in Jebel-Algana village. For Experiments 2 and 3, median values were calculated over 43 and 76 days follow-up respectively.

(a) Experiment 1

| Trap site | Trap type^1^ | Control (non-intervention) houses | | | | ODRS Treated houses | | | |
| --- | --- | --- | --- | --- | --- | --- | --- | --- | --- |
|  |  | Total *P. orientalis* | Male *P. orientalis* | Female *P. orientalis* | n traps nights | Total *P. orientalis* | Male *P. orientalis* | Female *P. orientalis* | n traps nights |
| **pre-intervention** |  |  |  |  |  |  |  |  |  |
| Inside huts | KD | 0.5 (0-1) | 0 (0-0) | 0.5 (0-1) | 8 | 1 (0-1.5) 0.9 | 0 (0-0) | 1 (0-1) | 8 |
| Outdoor | LT | 0 (0-1) | 0 (0-0) | 0 (0-0.5) | 32 | 1 (0-2) 1.3 | 0 (0-1) | 0 (0-2) | 32 |
| Outdoor | ST | 5.5 (2-10) | 2 (1-3) | 4 (1-8) | 32 | 8 (3.5-16.5) | 3 (1-7) | 5 (2-8.5) | 32 |
| Peridomestic | ST | 6 (3-7.5) | 1 (1-3) | 4 (2-5) | 32 | 9.5 (2-16.5) | 3 (1-8) | 5.5 (1-11) | 32 |
| sum |  | 2 (0.5-6.5) | 1 (0-2) | 2 (0-4) | 104 | 3 (1-11) | 1 (0-4) | 2 (0-6.5) | 104 |
|  |  |  |  |  |  |  |  |  |  |
| **post-intervention** |  |  |  |  |  |  |  |  |  |
| Inside huts | KD | 1.5 (1-2.5) | 0 (0-0.5) | 1 (0.5-2) | 8 | 0 (0-0.5) | 0 (0-0) | 0 (0-0.5) | 8 |
| Outdoor | LT | 0 (0-0) | 0 (0-0) | 0 (0-0) | 32 | 0 (0-0) | 0 (0-0) | 0 (0-0) | 32 |
| Outdoor | ST | 3.5 (1.5-5) | 1 (0.5-2) | 2 (1-3) | 32 | 0 (0-1) | 0 (0-0) | 0 (0-1) | 32 |
| Peridomestic | ST | 3 (2-5) | 1 (0-2) | 2 (1-4) | 32 | 0 (0-1) | 0 (0-0) | 0 (0-1) | 32 |
| sum |  | 2 (0-4) | 0 (0-1) | 1 (0-3) | 104 | 0 (0-1) | 0 (0-0) | 0 (0-1) | 104 |

1. Trap types: KD insecticide knockdown collection of *P. orientalis* inside sleeping huts; LT CDC light traps; ST sets of 10 sticky traps.

(b) Experiment 2

|  |  |  |  |  |  |  |  |  |  |
| --- | --- | --- | --- | --- | --- | --- | --- | --- | --- |
| Trap site | Trap type^1^ | Control (non-intervention) houses | | | | ODRS Treated houses | | | |
|  |  | Total *P. orientalis* | Male *P. orientalis* | Female *P. orientalis* | n traps nights | Total *P. orientalis* | Male *P. orientalis* | Female *P. orientalis* | n traps nights |
| **pre-intervention** |  |  |  |  |  |  |  |  |  |
| Outdoor | LT | 13 (10-30) | 11 (8-22.5) | 4 (2-6.5) | 16 | 21.5 (11-30.5) | 17.5 (9.5-23) | 4.5 (3-7.5) | 16 |
| Outdoor | ST | 14 (12-17) | 10 (9-12) | 4 (3-5) | 14 | 19 (10-30) | 14 (8-22) | 4.5 (2-7) | 14 |
| Peridomestic | ST | 19 (11-23) | 13.5 (9-17) | 5 (2-6) | 14 | 18.5 (16-27) | 13.5 (11-20) | 5.5 (3-7) | 14 |
| sum |  | 15 (11-23) | 11 (8.5-17.5) | 4 (2-6) | 44 | 19.5 (11-29) | 15 (9-21.5) | 5 (3-7) | 44 |
| **post-intervention** |  |  |  |  |  |  |  |  |  |
| Outdoor | LT | 16 (7-27) | 11 (6-20) | 4 (2-7) | 103 | 0 (0-0) | 0 (0-0) | 0 (0-0) | 103 |
| Outdoor | ST | 17 (10-28) | 14 (9-21.5) | 3 (2-5.5) | 76 | 0 (0-1) | 0 (0-1) | 0 (0-0) | 76 |
| Peridomestic | ST | 18 (9-29) | 14.5 (7-23.5) | 3 (2-6) | 76 | 0.5 (0-1) | 0 (0-1) | 0 (0-0) | 76 |
| sum |  | 17 (9-27) | 13 (7-21) | 3 (2-6) | 255 | 0 (0-1) | 0 (0-1) | 0 (0-0) | 255 |

1. Trap types: KD insecticide knockdown collection of *P. orientalis* inside sleeping huts; LT CDC light traps; ST sets of 10 sticky traps.

(c) Experiment 3

| Trap site | Trap type^1^ | Control (non-intervention) houses | | | | ODRS treated houses | | | | RODRS treated houses | | | |
| --- | --- | --- | --- | --- | --- | --- | --- | --- | --- | --- | --- | --- | --- |
|  |  | Total *P. orientalis* | Male *P. orientalis* | Female *P. orientalis* | n traps nights | Total *P. orientalis* | Male *P. orientalis* | Female *P. orientalis* | n traps nights | Total *P. orientalis* | Male *P. orientalis* | Female *P. orientalis* | n traps nights |
| **pre-intervention** |  |  |  |  |  |  |  |  |  |  |  |  |  |
| Outdoor | LT | 3.5 (2-6) | 2 (1-4) | 1 (0-3) | 32 | 3 (2-6) | 1.5 (1-3.5) | 2 (1-2) | 32 | 4 (2-6) | 2 (1-4) | 1.5 (1-3) | 32 |
| Outdoor | ST | 2 (1.5-5.5) | 1 (1-3) | 1 (0-2.5) | 32 | 3 (1.5-7.5) | 2 (1-4) | 1 (0-3) | 32 | 3 (2-6) | 2 (1-4) | 1.5 (1-2.5) | 32 |
| Peridomestic | ST | 3 (1-4.5) | 1 (0.5-2) | 1 (0-3) | 32 | 3.5 (2-6.5) | 2 (1-3.5) | 1.5 (1-3) | 32 | 3 (0.5-5.5) | 1 (0-2) | 1 (0-3) | 32 |
| sum |  | 3 (2-6) | 1 (1-3) | 1 (0-3) | 96 | 3 (2-7) | 2 (1-4) | 1.5 (1-3) | 96 | 3 (1.5-6) | 2 (1-4) | 1 (0.5-3) | 96 |
| **post-intervention** |  |  |  |  |  |  |  |  |  |  |  |  |  |
| Outdoor | LT | 1 (0-2) | 0 (0-1) | 0 (0-1) | 83 | 0 (0-1) | 0 (0-0) | 0 (0-0) | 85 | 0 (0-1) | 0 (0-1) | 0 (0-0) | 85 |
| Outdoor | ST | 2 (0-3) | 1 (0-2) | 0 (0-2) | 80 | 0 (0-1) | 0 (0-1) | 0 (0-0) | 79 | 1 (0-2.5) | 0 (0-1) | 0 (0-1) | 80 |
| Peridomestic | ST | 1.5 (1-4) | 1 (0-2) | 1 (0-2) | 80 | 0 (0-1) | 0 (0-1) | 0 (0-1) | 79 | 1 (0-2) | 0 (0-1) | 0 (0-1) | 80 |
| sum |  | 2 (0-3) | 1 (0-2) | 1 (0-1) | 243 | 0 (0-1) | 0 (0-1) | 0 (0-0) | 243 | 1 (0-2) | 0 (0-1) | 0 (0-1) | 245 |

1. Trap types: KD insecticide knockdown collection of *P. orientalis* inside sleeping huts; LT CDC light traps; ST sets of 10 sticky traps.
