# Supplementary material for: Outdoor Residual Insecticide Spraying (ODRS), a New Approach for the Control of the Exophilic Vectors of Human Visceral Leishmaniasis: Phlebotomus orientalis in East Africa
Source: PLoS Negl Trop Dis. 2020 Oct 20;14(10):e0008774. doi: 10.1371/journal.pntd.0008774 (PMC7598920; doi:10.1371/journal.pntd.0008774)
Supplement: Attachment — Submitted filename: P orientalis control.pdf [file pntd.0008774.s003.pdf]

PLOS Neglected Tropical Diseases

Outdoor Residual Insecticide Spraying, a New Approach for the Control of the Exophilic Vectors of Human Visceral Leishmaniasis: Phlebotomus orientalis in East Africa

--Manuscript Draft--

**Manuscript Number:**	PNTD-D-20-00991
**Full Title:**	Outdoor Residual Insecticide Spraying, a New Approach for the Control of the Exophilic Vectors of Human Visceral Leishmaniasis: Phlebotomus orientalis in East Africa
**Short Title:**	Outdoor residual spraying insecticide to control sand fly vectors
**Article Type:**	Research Article
**Keywords:**	Phlebotomus orientalis; Leishmania donovani; leishmaniasis, vector control; outdoor residual spraying; insecticide; cost-effectiveness; Sudan.
**Abstract:**	Background . Visceral Leishmaniasis (VL) due to Leishmania donovani is a neglected protozoan parasitic disease in humans, which is usually fatal if untreated. Phlebotomus orientalis , the predominant VL vector in East Africa, is a highly exophilic/exophagic species that poses a major challenge to current Integrated Vector Management (IVM). Here we report results of pilot studies conducted in rural villages in Gedarif state, Sudan, to evaluate outdoor residual spraying of 20mg active ingredient (a.i.) /m ^{2} deltamethrin insecticide applied to the characteristic household compound boundary reed fence and to the outside of household buildings (ODRS), and as an alternative, spraying restricted to the boundary fence only (RODRS).<br>Methods . Four to six clusters of 20 households were assigned to insecticide treatments or control in three experiments. Changes in sand fly numbers were monitored over 2,033 trap-nights over 43-76 days follow-up in four sentinel houses per cluster relative to unsprayed control clusters. Sand fly numbers were monitored by sticky traps placed on the ground on the inside (“outdoor”) and the outside (“peridomestic”) of the boundary fence, and by CDC light trap suspended outdoors in the household compound. Intervention effects on sand fly numbers inside sleeping huts were monitored by insecticide knockdown.<br>Results . After a single application, ODRS reduced P. orientalis abundance by 83%-99% in outdoor and peridomestic trap locations. ODRS also reduced numbers of P. orientalis found resting inside sleeping huts. RODRS reduced outdoor and peridomestic P. orientalis by 60%-88%. By direct comparison, RODRS was 58%-100% as effective as ODRS depending on the trapping method. These impacts were immediate on intervention and persisted during follow-up, representing a large fraction of the P. orientalis activity season. Relative costs of ODRS and RODRS delivery were $5.76 and $3.48 per household, respectively.<br>Conclusions . The study demonstrates the feasibility, high entomological efficacy, and expected low costs relative to current IVM practises. ODRS and RODRS is a novel sand fly vector control method against exophilic/exophagic sand fly vectors to lower VL burdens in Sudan, with potential application in other endemic regions in East Africa.
**Additional Information:**	
**Question**	**Response**
**Financial Disclosure**<br><br>Enter a financial disclosure statement that describes the sources of funding for the work included in this submission. Review the submission guidelines for detailed requirements. View published research articles from *PLOS NTDs* for specific examples.	The authors gratefully acknowledge financial support from Department of International Development (DFID), the UK AID programme, on Tackling Visceral Leishmaniasis in East Africa, through the KalaCORE consortium (contract PO 6361) (www.kalacore.org). The funders had no role in study design, data collection and analysis, decision to publish, or preparation of the manuscript. The views expressed do not necessarily reflect the UK government’s official policiesThe funders had no role in study design, data collection and analysis, decision to publish, or preparation of the manuscript.

Powered by Editorial Manager® and ProduXion Manager® from Aries Systems Corporation

This statement is required for submission and **will appear in the published article** if the submission is accepted. Please make sure it is accurate.<br><br>**Unfunded studies**<br><br>Enter: *The author(s) received no specific funding for this work.*<br><br>**Funded studies**<br><br>Enter a statement with the following details:<br>• Initials of the authors who received each award<br>• Grant numbers awarded to each author<br>• The full name of each funder<br>• URL of each funder website<br>• Did the sponsors or funders play any role in the study design, data collection and analysis, decision to publish, or preparation of the manuscript?<br>• **NO** - Include this sentence at the end of your statement: *The funders had no role in study design, data collection and analysis, decision to publish, or preparation of the manuscript.*<br>• **YES** - Specify the role(s) played.<br><br>* typeset	
**Competing Interests**<br><br>Use the instructions below to enter a competing interest statement for this submission. On behalf of all authors, disclose any competing interests that could be perceived to bias this work—acknowledging all financial support and any other relevant financial or non-financial competing interests.<br><br>This statement **will appear in the**	The authors have declared that no competing interests exist

**published article** if the submission is accepted. Please make sure it is accurate. View published research articles from *PLOS NTDs* for specific examples.<br><br>**NO authors have competing interests**<br><br>Enter: *The authors have declared that no competing interests exist.*<br><br>**Authors with competing interests**<br><br>Enter competing interest details beginning with this statement:<br><br>*I have read the journal's policy and the authors of this manuscript have the following competing interests: [insert competing interests here]*<br><br>* typeset	
**Data Availability**<br><br>Authors are required to make all data underlying the findings described fully available, without restriction, and from the time of publication. PLOS allows rare exceptions to address legal and ethical concerns. See the PLOS Data Policy and FAQ for detailed information.<br><br>A Data Availability Statement describing where the data can be found is required at submission. Your answers to this question constitute the Data Availability Statement and **will be published in the article**, if accepted.	Yes - all data are fully available without restriction

**Important:** Stating 'data available on request from the author' is not sufficient. If your data are only available upon request, select 'No' for the first question and explain your exceptional situation in the text box.<br><br>Do the authors confirm that all data underlying the findings described in their manuscript are fully available without restriction?	
**Describe where the data may be found in full sentences. If you are copying our sample text, replace any instances of XXX with the appropriate details.**<br><br>• If the data are **held or will be held in a public repository**, include URLs, accession numbers or DOIs. If this information will only be available after acceptance, indicate this by ticking the box below. For example: *All XXX files are available from the XXX database (accession number(s) XXX, XXX.*).<br>• If the data are all contained **within the manuscript and/or Supporting Information files**, enter the following: *All relevant data are within the manuscript and its Supporting Information files.*<br>• If neither of these applies but you are able to provide **details of access elsewhere**, with or without limitations, please do so. For example:<br><br>*Data cannot be shared publicly because of [XXX]. Data are available from the XXX Institutional Data Access / Ethics Committee (contact via XXX) for researchers who meet the criteria for access to confidential data.*	All relevant data are within the manuscript and its Supporting Information files.

Powered by Editorial Manager® and ProduXion Manager® from Aries Systems Corporation

The data underlying the results presented in the study are available from (include the name of the third party and contact information or URL).<br>• This text is appropriate if the data are owned by a third party and authors do not have permission to share the data.<br><br>* typeset	
Additional data availability information:	

Powered by Editorial Manager® and ProduXion Manager® from Aries Systems Corporation

Manuscript Text
Click here to download Manuscript Elnaiem-et-al-ODRS-MS-final-Jun-4-2020 (1).docx

Running head: Outdoor residual spraying insecticide to reduce sand fly vectors

Outdoor Residual Insecticide Spraying, a New Approach for the Control of the Exophilic Vectors of Human Visceral Leishmaniasis: *Phlebotomus orientalis* in East Africa

Dia-Eldin A Elnaiem[1]*, Osman Dakein[2,3], Ahmed Ali Elawad[4,5], Bashir Alsharif[6], Eltayieb Khogali[5], Taiseer Gibreel[5], Omran F Osman[2], Hassan Has'san[4,5], Atia Mohamed Atia[7], Mousab S Elhag[8], Margriet Den Boer[9], Koert Ritmeijer[9], Caryn Bern[10], Jorge Alvar[11], Noteila Khalid[12], and Orin Courtenay[13]*

Affiliations

[1] Department of Natural Sciences, University of Maryland Eastern Shore, MD 21853.

[2] Department of Zoology, Faculty of Science, University of Khartoum.

[3] Kala azar Research Centre, Faculty of Medicine and Health Sciences, University for Gedarif, Gedarif, Sudan

[4] Ministry of Health, Gedarif state, Sudan.

[5] Sudan Blue Nile Health Institute, Gezira University, Wad Medani,

[6] Departamento de Entomologia, CPqAM, Fundação Oswaldo Cruz, Recife, Brasil and Dept of Medical Entomology, National Medical Laboratory, Ministry of Health, Sudan.

[7] ASCEND Program, Khartoum, Sudan

[8] Director, Directorate of Communicable Diseases, Federal Ministry of Health, Khartoum, Sudan

[9] Medecins Sans Frontieres, Amsterdam, The Netherlands

[10] University of California San Francisco, San Francisco, California, United States of America.

[11] Drugs for Neglected Diseases *initiative*, Geneva, Switzerland

[12] Department of Zoology, Ibn Sina University, Khartoum, Sudan.

[13] Zeeman Institute and School of Life Sciences, University of Warwick, Coventry, CV4 7AL, UK.

*Corresponding authors

daelnaiem@umes.edu

orin.courtenay@warwick.ac.uk

ORCID numbers

DE: 0000-0002-3651-7904

OC: 0000-0002-0188-6929

JA: 0000-003-2920-742X

MB: 0000-0003-1598-2206

KR: 0000-0001-5924-872X

CB: 0000-0001-8195-7303

EK: 0000-0001-6519-7607

AE: 0000-0002-7705-7617

HH: 0000-0002-3114-6358

DO : 0000-0001-5713-8551

NK: 0000-0001-7333-0973

BA: 0000-0002-9974-3503

ME: 0000-0001-6212-8243

OO: 0000-0001-5056-6561

AA: 0000-0002-8452-7891

Key words: *Phlebotomus orientalis*; *Leishmania donovani*; leishmaniasis, vector control; outdoor residual spraying; insecticide; cost-effectiveness; Sudan.

Abstract

Background. Visceral Leishmaniasis (VL) due to *Leishmania donovani* is a neglected protozoan parasitic disease in humans, which is usually fatal if untreated. *Phlebotomus orientalis*, the predominant VL vector in East Africa, is a highly exophilic/exophagic species that poses a major challenge to current Integrated Vector Management (IVM). Here we report results of pilot studies conducted in rural villages in Gedarif state, Sudan, to evaluate outdoor residual spraying of 20mg active ingredient (a.i.) /m^2 deltamethrin insecticide applied to the characteristic household compound boundary reed fence and to the outside of household buildings (ODRS), and as an alternative, spraying restricted to the boundary fence only (RODRS).

Methods. Four to six clusters of 20 households were assigned to insecticide treatments or control in three experiments. Changes in sand fly numbers were monitored over 2,033 trap-nights over 43-76 days follow-up in four sentinel houses per cluster relative to unsprayed control clusters. Sand fly numbers were monitored by sticky traps placed on the ground on the inside (“outdoor”) and the outside (“peridomestic”) of the boundary fence, and by CDC light trap suspended outdoors in the household compound. Interven[illegible] effects on sand fly numbers inside sleeping huts were monitored by insecticide knockdown.

Results. After a single application, ODRS reduced *P. orientalis* abundance by 83%-99% in outdoor and peridomestic trap locations. ODRS also reduced numbers of *P. orientalis* found resting inside sleeping huts. RODRS reduced outdoor and peridomestic *P. orientalis* by 60%-88%. By direct comparison, RODRS was 58%-100% as effective as ODRS depending on the trapping method. These impacts were immediate on intervention and persisted during follow-up,

representing a large fraction of the *P. orientalis* activity season. Relative costs of ODRS and RODRS delivery were $5.76 and $3.48 per household, respectively.

Conclusions. The study demonstrates the feasibility, high entomological efficacy, and expected low costs relative to current IVM practises. ODRS and RODRS is a novel sand fly vector control method against exophilic/exophagic sand fly vectors to lower VL burdens in Sudan, with potential application in other endemic regions in East Africa.

Authors' summary

Phlebotomus orientalis is the predominant vector of visceral leishmaniasis (VL, kala azar), in Sudan and other countries of East Africa, where the disease causes high morbidity and mortality. This sylvatic sand fly species is abundant in wild habitats characterized by presence of black cotton soil and vegetation dominated by *Balanites aegyptiaca* and/or *Acacia seyal* trees. In villages, the vector bites people in the household yard and in nearby peri-domestic locations, exhibiting limited indoor resting behaviour. The marked exophagic and exophilic behaviours of *P. orientalis* represent a profound challenge for VL control by excluding indoor residual spraying of insecticides (IRS) and compromising the efficacy of insecticide-impregnated bednets (ITNs). In this study, we evaluated the entomological efficacy of residual pyrethroid applied outdoors to household boundary fences and the exterior walls of household huts (ODRS), to reduce the abundance of *P. orientalis* inside and outside houses. We also evaluated the entomological impact of a restricted version of outdoor spraying (RODRS), whereby insecticide was applied only to the boundary fence. The study was carried out in June 2016-June 2017 in Jebel-Algana and Umsalala villages, Gedarif state, eastern Sudan, which are highly endemic for VL. The results showed that a single ODRS application of 20mg a.i. /m^2 2.8% deltamethrin

provided average reductions of 83%-99% in outdoor and peridomestic *P. orientalis* sand fly numbers relative to unsprayed control clusters. RODRS reduced outdoor and peridomestic *P. orientalis* by 60%-88%. The average cost of ODRS and RODRS per household were $5.76 and $3.48, respectively. The costs of these community-based control measures were substantially lower than the costs of LLINs, which is the only evidence-based tool used to protect against VL in the area. Future studies should evaluate the impact of ODRS/RODRS transmission of VL incidence in endemic villages and in seasonal agricultural farms.

Introduction

Standard methods to combat hematophagous arthropod vectors of infectious diseases include – among other possibilities- indoor residual spraying of insecticides (IRS) and use of insecticide-treated bednets (ITNs), the success of which largely rely on insecticide-susceptible vector populations showing endophilic and/or endophagic blood-feeding behaviours. Sustainable methods to control exophilic/exophagic vectors, by contrast, are lacking, but key in the fight against many of the so-called "tool-deficient" Neglected Tropical Diseases (NTDs) [1,2,3]. One such challenge includes the Phlebotomine sand fly vectors of the protozoan parasites *Leishmania* causing human visceral leishmaniasis (VL, also known as kala azar) which occurs in East Africa, the Indian sub-continent, the Americas, the Mediterranean region, and Central and Eastern Asia. Globally, an underreported estimated 31,000-90,000 new cases are reported each year [4,5].

Visceral leishmaniasis, caused by *L. donovani* is a serious public health problem in E. Africa with some of the highest case incidences worldwide resulting in severe epidemics [4, 5,6,7,8,9,10,11,12], and exerting tremendous social and economic burdens on afflicted populations [13,14]. The symptoms of systemic clinical VL development include fever, weight

loss, fatigue, splenomegaly and hepatomegaly. VL is usually fatal within two years if not treated, and there is no ~~human~~ vaccine.

In East Africa, the ~~prominent~~ VL vector is *Phlebotomus orientalis*, which thrives in remote woodlands, villages, and peridomestic habitats, characterized by the presence of black cotton soil and *Acacia seyal* and *Balanites aegyptiaca* trees [13,15,16,17,18,19,20,21]. The vector is highly exophilic and exophagic, rarely captured inside household buildings [22,23,24,25,26,27,28] with villagers being exposed outdoors in the household compound and in the peridomestic surroundings.

The highest burden of clinical VL in East Africa occurs in Sudan and South Sudan [4,5] with ~~highest~~ prevalence (43%-70%) reported in children <15 year old [4,12,29]. In Sudan, 90% of cases occur in Gedarif state, eastern Sudan, where 51,200 patients were registered receiving treatment against VL between 2002-2015 [12], and case incidence ranges between 26–37/1000/year (2014–2017 MoH data) resulting in 2,000-7,000 newly reported cases per year [12,30].

Integrated Vector Management (IVM) recommendations in the region include IRS using pyrethroid or Bendiocarb – (a carbamate)- twice per year, and provision of long-lasting insecticide-impregnated bednets (LLINs) to villagers. These approaches have been shown to impact on the local mosquito vectors of malaria e.g. *Anopheles arabiensis* [31], which bites and rest inside huts during the rainy season. In contrast, IRS has little potential in control of exophilic and exophagic species such as *P. orientalis*, which is unlikely to land on unsprayed or sprayed ~~indoor~~ wall surfaces [32]. Furthermore, the effectiveness of LLINs against *P. orientalis* is compromised by the habit of ~~sleeping~~ outdoors in the uncomfortably hot weather during the sand fly season (requiring setting up the bednets each night), and where a large fraction of *P.*

orientalis bites occur early evening, before bedtime [9]. Indeed, the propensity to sleep outdoors may be a contributory factor to the marked exophagic behaviour of *P. orientalis* in Sudan and elsewhere.

To provide privacy to this outdoor sleeping area, each household yard is surrounded by 1.5-2 m tall fence made of tightly thatched reed, thus separating the outdoor yard from the immediate peridomestic area. In a recent investigation of microhabitat distribution of *P. orientalis* in 6 villages in Gedarif state, we captured lower numbers of the vector inside houses than their immediate surrounding; a result indicating that these boundary fences act as physical barriers, reducing numbers of *P. orientalis* entering the household yard and sleeping huts from the peridomestic habitats [33].

In pursuit of feasible alternative community-wide vector control methods, we investigated the impact on vector abundance of outdoor coverage with residual pyrethroid insecticide. Pilot studies were designed specifically to test the en[illegible]ological efficacy of (i) outdoor residual spraying of the walls of household huts and the boundary fence (ODRS), to reduce indoor, outdoor and peridomestic *P. orientalis* abundance; (ii) to compare the efficacy of ODRS to an alternative insecticide application restricted to spraying boundary fences alone (RODRS); and (iii) to estimate the relative costs of ODRS and RODRS.

Methods

Study sites

The study was carried out in the rural villages of Jebel-Algana (13.6294444, 36.13666667) and Umsalala (12.85055556, 35.1822222) in Gedarif State, eastern Sudan, located within 70km of

and 21.9% in the winter. *Phlebotomus orientalis* is active for 3-4 months between March and June, being most abundant at the end of the hot dry season, after which the abundance rapidly declines at the onset of the heavy seasonal rains in June/July [15,17].

The resident populations of Jabel-Algana (3,955 inhabitants) and Umsalala (2,710 inhabitants) are predominantly agrarian comprising a number of ethnic groups following waves of migration for agricultural work between the early 1950s to the 1980s from the Darfur region, western Sudan [34]. Household compounds are typically surrounded by a 1.5-2 metre tall boundary fence made of tightly woven thatched reed *Cymbogon nyrvatus*, primarily to provide privacy (Figure 2A). Compounds contain an average of three (range: 1-4) huts ("rakobas"), one used for keeping belongings and sleeping in cooler months; the others used for cooking, storage, or daytime shelter. These huts are predominantly constructed of a wooden frame thatched with grass on the walls and roof. Most households maintain a variable small number of mixed livestock (chickens, sheep, goats, cattle, and donkeys); villagers rarely keep dogs or cats. Due to the excessive heat, people sleep outside in the compound throughout October–May, and on hotter dry nights of June-September [9,38]. They sleep inside huts only during the rains (June-October) and in the winter (November- February). The villages are designed on a grid where blocks of contiguous houses are separated from neighbouring blocks by well-demarcated earthen tracks (Figure 2B).

Fig. 2. Images of typical household layout in VL endemic villages in Gedarif state, eastern Sudan (this example in Jebel-Algana study village). Each house consists of 2-4 thatched grass huts surrounded by a tall thatched reed fence that provides privacy. (A) *Acacia* and *Balanites* trees, associated with *P. orientalis* vectors, are commonly present within the property boundary,

the northern Sudanese-Ethiopian border (Figure 1). Jebel-Algana is in Eastern Gureisha Mahlia (district), near the Atbara River, 129 km distance from Umsalala village [Western Galabat Mahalyia], located on the eastern bank of the River Rahad.

Fig. 1. Map of Gedarif state, showing location of Jebel-Algana and Umsalala study villages in relation to Tabarakllah and Umelkher kala azar treatment centers.

The villages are representative of this hyperendemic region of VL in eastern Sudan [34], experiencing high transmission rates with VL case incidence of 26–37/1000/year at the time of the study (2014–2017 Hospital Records; Ministry of Health [MoH] unpublished data). The regional stability in VL incidence, indicating stability in *P. orientalis* biting rates, is reflected in similarities to historical high VL annual incidence [35,36,37,38]

Both villages lie in an area that was previously covered by dense woodlands of *Acacia seyal* and ~~*Balanitis*~~ *aegyptiaca*, but now greatly reduced by mechanized seasonal plantations of sorghum, sesame, pearl millet (“dokhun”) and groundnuts, leaving remnant thickets of *B. aegyptiaca, A. seyal* and *Zyzyphus spina christi* trees in and around the villages. The soil is predominantly black cotton soil (chromic vertisols), interrupted by sandy and silt soils (“azaza”). During the dry season, the black cotton soil shrinks; creating deep cracks which are thought to harbour several species of sand fly including *P. orientalis* [15,18,39]. The climate is characterised as tropical continental, with annual rainfall of approximately 600-800 mm. The year is divided into a hot dry season (March-May), a moderately warm rainy season (June-October), and a warm dry winter (November- February). The regional mean temperature and relative humidity respectively are 32.3°C and 25.9% in the hot dry season, 26.2°C and 54.4% in the rainy season, and 27.0°C

and (B) in the narrow earthen streets that separate household blocks. During the VL transmission season people sleep outside in within the household yard.

Village, cluster and sentinel house recruitment

Villages. The two study villages were selected as a convenience sample from many possible VL endemic villages along the Atbara and Rahad rivers, on the criteria that within 1-2 years prior to the study there was evidence of active *L. donovani* transmission; an abundance of *P. orientalis* in the villages; and they were geographically located within 5 km from the field operations centres in Tabarakallah and Umelkher, respectively, to aid in logistical requirements. Jebel-Algana and Umsalala villages had 17 and 55 human VL cases recorded by the nearby Kala-azar treatment centres in Tabarakallah (data for 2015-2016) and Umelkher (data for 2016-2017), respectively. *Phlebotomus orientalis* presence was demonstrated by sand fly capture conducted in 2015 -2016 [33]. During the current study, as in past entomological surveys, *P. orientalis* densities were generally higher in Umsalala than in Jebel-Algana [33].

Clusters. Clusters were defined as 20 contiguous households. Ten clusters were identified in Jebel-Algana and Umsalala villages for possible recruitment into the study. The selection criteria included that clusters within villages were separated from neighbouring clusters by a distance of >300 metres to avoid possible contamination by sand fly dispersal [40,41,42]; that clusters showed relatively typical vegetation, topography, soil type and that each of the household compounds was surrounded by the characteristic reed fence.

Following consultation with village leaders, and informed written consent from all householders, four clusters in Jebel-Algana village (Experiment 1), and four clusters in Umsalala villages (Experiment 2) were recruited. The same four clusters used in Experiment 1 in 2016, plus two additional clusters in the same village, Jebel-Algana, were recruited for Experiment 3 in 2017. Due to the grid design of houses within villages, each household faced an earthen street on one side, and bordered neighbouring household compounds on the other sides. Therefore, to maximise proximity of households within clusters, each cluster comprised one linear block of 10 contiguous houses separated from the second block of 10 contiguous houses by a narrow earthen street (2-3 metres wide).

Sentinel houses. Four sentinel households per cluster were identified for monitoring intervention effects on sand fly abundance. The four households were located in the centre of the cluster i.e. two households in the centre of each block of ten houses. The portion of the boundary fence facing the earthen street were the focus for monitoring sand fly numbers as described below.

Study design

The study comprised of three experiments: Experiment 1, conducted between 9th and 16th June 2016, was the initial scoping study to assess the potential and feasibility of ODRS to reduce *P. orientalis* abundance inside sleeping huts, outdoors in the household compound, and outside the compound boundary fence. Experiment 2 was designed to evaluate the residual effectiveness of a single application of ODRS against outdoor and peridomestic sand fly numbers compared to controls followed-up over 43 days post intervention. Experiment 3 similarly aimed to assess the residual effectiveness against *P. orientalis* numbers of a single ODRS application, compared to single restricted ODRS (RODRS) application; over 76 follow-up days post intervention.

Experiments 2 and 3 were conducted between 2nd May and 16th June 2017, and between 31st March and 20th June 2017, respectively. Following the baseline pre-intervention sample, each intervention was applied on a single occasion per experiment, on 13h June 2016 (Experiment 1), 4th May 2017 (Experiment 2), and 5th April 2017 (Experiment 3).

Intervention randomization

The enrolled clusters were randomised to one of the two interventions (Experiments 1 & 2), or to one of three interventions (Experiment 3), by an independent blinded local observer who selected folded slips of paper with cluster names written on them from a container. The interventions were assigned alternatively in order that cluster names were drawn to achieve two clusters per treatment in each Experiment 1-3. All houses within a cluster received the same intervention. No significant differences in pre-intervention fly numbers captured by Sticky traps (ST) or CDC light trap (LT) were detected when controlling for study design variables (see below), suggesting that the randomisation process for the three Experiments was successful ($z<2.01$, $p>0.347$).

The interventions

ODRS comprised of spraying 2.8% deltamethrin WP insecticide (Scientific Fertiliser Co. PVT. Ltd, Mysuru, Puram Colinbatore, India) at a dose of 20mg a.i. /m^2 on the exterior walls of all huts (“rakobas”), and on both the interior and exterior surface of the household compound boundary fence. Restricted ODRS (RODRS), comprised of spraying only the boundary fence as described above. Control households and fences were left unsprayed.

Insecticide was applied using a 10 litre Hudson spray pump (Hudson Products, X-Pert 67322AD Professional Sprayer, Hudson Manufacturing Company, Hong Kong). The tank solution was

prepared by one trained technician, and the insecticide applied by three trained Gedarif health authority personnel responsible for routine IRS under the regional IVM program. The interventions were supervised by the research team. All spraying personnel wore appropriate protective clothing, goggles and masks.

Sand fly sampling

Sand fly traps were placed pre- and post-intervention in the four sentinel households per cluster. Sticky traps (ST) were made of standard A4 sheets of white paper coated with sesame oil. Sets of 10 sticky traps were laid horizontally (sticky-side up) on the ground in a line spaced 0.5 m apart; one set was positioned 30cm from the inside of the household compound boundary fence (labelled “outdoor” trap location). Another set of 10 STs were similarly positioned on the outside of the boundary fence (labelled “peridomestic” trap location) that faced the earthen street. STs were in position from 18:00 hrs and collected the following morning at 06:00 hrs. In addition, a single CDC light trap (LT) (Model 512, John Hock Company, USA) without the bulb was set outdoors within the household compound, positioned approximately 1 meter from the compound boundary fence and suspended 0.5 meters from the ground from a tree branch or bamboo pole. LT placement and collection times were the same as for the STs.

A standard chemical knockdown (KD) technique [17] was used to determine the numbers of sand flies resting inside the household sleeping hut (Experiment 1 only). Between 06:00-07:00 hrs, the floor was covered with white cotton sheets, the room tightly sealed, and then spatially sprayed with 250 ml combination of 0.2% Tetramethirn, 0.025% Cyluthrin, 1.0% Piperonyl

Butoxide, and 98.8% solvents and propellants (Flytex, Khartoum, Sudan). Fifteen minutes post spatial spraying; sand flies were carefully collected from the floor sheets for identification.

Sand fly identification

Collected sand flies were washed in mild detergent solution, rinsed in distilled water and immediately stored in Eppendorf tubes containing 70% ethanol. Sand flies were initially sorted under a dissecting microscope to separate *Phlebotomus* from *Sergentomyia spp*. *Phlebotomus* specimens were then individually mounted in PVA medium (BioQuipp, USA) to inspect the spermatheca, pharyngeal armature and male genitalia under a binocular microscope to confirm species identification following relevant taxonomic keys [43, 15].

Statistical analysis

The unit of study was the cluster-level *P. orientalis* trap count. Trap counts of male and female *P. orientalis* combined were standardised to represent numbers captured per trap per night ("trap-night"). The sum number caught by one set of 10 STs per night, or by one CDC light trap per night, was considered a trap-night, respectively.

The intervention effects in treated compared to in control clusters were estimated by fitting the standardised trap counts to negative binomial regression models, including variables describing the experimental design structure as appropriate including trap-type code, trap-site code, number of trap-nights, days from intervention, and a quadratic term ($days^2$) to account for non-linearity in outcome measures over follow-up time (Experiments 2 and 3). Study cluster ID was entered as a cluster term in the model, and pre-intervention (baseline) log_{10}-transformed (+1) number of *P. orientalis* was used as the model offset parameter.

The potential variability in intervention effects between trap types and trap sites, and time (days and $days^2$) from intervention, were examined by model inclusion and testing of relevant interaction term(s). Effect estimates were expressed as incidence risk ratio (IRR) generated using post-estimation routines (LINCOM) in STATA.

Data from Experiments 2 and 3 were fitted to equivalent mixed effects (ME) models where cluster ID was treated as a random effect term, but this did not improve the model fits (Log-likelihood Ratio Test: $p>0.05$), or the models failed to converge.

Data were analyzed using Stata v.15.1 software (StataCorp LP, College Station, TX).

Insecticide delivery cost estimation

The costs of insecticide use for ODRS and RODRS were calculated based on the average inner and outer surface boundary fence area to be sprayed estimated to be $175m^2$ by measuring the convenience sample of 40 households treated in Experiment 1. For ODRS, this was added to the exterior wall surface area of 3 huts per household using a previously estimated average surface area of a single hut of 49.17 m^2 (Gedarif state IRS technical manual, unpublished). Insecticide usage for each of the 40 households was also recorded. The average costs of ODRS and RODRS per household were then calculated including the *pro rata* costs of skilled labour for three trained labourers (sprayers) and one technician (to prepare the Hudson tank solution) as practised in this study. The labour time to deliver RODRS was an average 30% less than that to deliver ODRS. For reporting, the total costs were converted using the 2016 official exchange rate of $1 USD = 6.6 Sudanese pounds (SDG).

Ethical Considerations

Insecticide spraying was approved by the district (Mahalia) health authorities of Gureisha and Rahad. Following a full explanation of the spraying procedures and the study objectives, informed written consent was obtained from the heads of all recruited households, and the head of the popular committee of the village. The study did not involve sampling human subjects.

RESULTS

Trapping efficiency

Across the three experiments, a total of 10,813 *P. orientalis* sand flies were captured in 2,033 trap-nights, of which 33% were female flies (Table 1). Trap counts in clusters not treated with insecticide (pre- and post-intervention) indicated that households sampled in Experiment 2, conducted in Umsalala village (in 2017), produced substantially higher *P. orientalis* trap counts than in Experiment 1 (in 2016) or in Experiment 3 (in 2017), both conducted in Jebel-Algana (Figure 3; Table S1; Table S2).

Fig. 3. Differences in natural vector abundance between Experiments 1-3 as measured in households not treated with insecticide. Median numbers of *P. orientalis* captured per trap-night in households pre-treatment (pre-T) and post-treatment (post-T), in control (C), ODRS and RODRS households. Experiments: Exp.1: black bars; Exp. 2: grey bars; Exp 3. white bars. For consistency across experiments, values were calculated excluding KD captures (made only in Experiment 1). Error bars represent the 25th to 75th interquartile range.

Differences in LT and ST counts ("trapping efficiency") within and between experiments were also variable (Table 1; Table S2), though controlling for covariates, no consistent differences in

the mean numbers captured by the two methods were detected pre-intervention cluster samples (Z=0.19, P=0.846, N=568), or in post-intervention control cluster samples (Z=0.58, P=0.564, N=594). However due to inherent differences between ST and LT mechanisms, the intervention effects on sand fly numbers were calculated separately for each trap type (thus trap location).

Table 1. Summary of the *P. orientalis* sand fly counts pre- and post-intervention in sentinel households across the three experiments.

Trap site	Trap type[1]	Total *P. orientalis*	Male *P. orientalis*	Female *P. orientalis*	n trap nights[1]
Pre-intervention Controls					
Inside sleeping huts	KD	4	0	4	8
Outdoor	LT	480	346	134	80
Outdoor	ST	531	283	248	78
Peridomestic	ST	560	298	262	78
Sum		1575	927	648	244
Pre-intervention Treated ODRS/RODRS					
Inside sleeping huts	KD	7	1	6	8
Outdoor	LT	697	455	242	112
Outdoor	ST	1022	563	459	110
Peridomestic	ST	907	486	421	110
Sum		2633	1505	1128	340

Post-intervention Controls					
Inside sleeping huts	KD	13	3	10	8
Outdoor	LT	2056	1539	517	218
Outdoor	ST	1825	1364	461	188
Peridomestic	ST	1905	1428	477	188
Sum		5799	4334	1465	602
Post-intervention Treated ODRS/RODRS					
Inside sleeping huts	KD	2	0	2	8
Outdoor	LT	162	111	51	305
Outdoor	ST	334	195	139	267
Peridomestic	ST	308	177	131	267
Sum		806	483	323	847
Totals		10813	7249	3564	2033

[1] total numbers of trap nights represent the sum of chemical knockdown (KD) events inside sleeping huts; a single CDC light trap (LT) set per household at outdoor sites per night, and a set of 10 sticky traps (ST) set at each outdoor and peridomestic site per night. Each set of 10 STs is considered a single trap-night.

Experiment 1

The initial experiment was conducted to assess the feasibility of ODRS as a vector control option. *P. orientalis* were trapped 1-4 days pre-ODRS, and 1-4 days post-ODRS application. Accounting for study design characteristics, *P. orientalis* abundance was reduced by 83% (95%

CL: 70.1%-90.9%) and 87% (95% CL: 76.3%, 93.0%) at outside and peridomestic ST locations, respectively, relative to in control clusters (Table 2, Figure 4).

Table 2. Intervention (ODRS) effect estimates on *P. orientalis* sand fly vector catches conducted over 8 days, by trap type and trap location.

Trap type	Trap location	IRR (95% C.L.)	P<	N Trap nights
ST	O	0.17 (0.091, 0.299)	0.0001	128
ST	P	0.13 (0.070, 0.237)	0.0001	128

Trap types for monitoring: ST sticky traps
Trap site locations: O outdoors, P peridomestic.

Fig. 4. Median number of *P. orientalis* captured per trap night in Experiment 1. Numbers in ODRS-treated (dark grey bars) and control (light grey bars) households trapped 1-4 days pre-ODRS (X-axis label -4 days), and 1-4 days post-ODRS application (X-axis label 4 days), measured by sticky traps set (A) at outdoor sites, and (B) at peridomestic sites. Error bars represent the IQR (25-75 percentiles). The experiment was conducted from the 9th to 16th of June 201[illegible].

ST captures at outdoor and peridomestic trap locations were not dissimilar (test of intervention × trap site interaction term: z=1.53, p=0.125, N=256 trap nights) (Table 2).

P. orientalis captures in sleeping huts collected by chemical knockdown (KD), and by LTs in outdoor locations, were few but showed significant reductions: inside huts, 4 and 7 specimens were collected in control and ODRS clusters respectively by pre-intervention sample, *versus* 13 and 2 specimens post intervention (Fisher's exact: p=0.014; N=16 trap nights). In outdoor LTs, 17 and 42 specimens were collected pre-intervention compared to 7 and 0 flies post-intervention (Fisher's Exact, P<0.001, N=16 trap nights).

Experiment 2

The effects of a single ODRS application over 43 days follow-up was assessed. ODRS reduced ST numbers by an average 96% (95% CL: 92.0%, 97.2%) and 92% (95% CL: 88.7%, 94.9%) in outdoor and peridomestic locations, respectively, and by 99% (95% CL: 99.2%, 99.5%) in LT outdoor locations, relative to control clusters (Table 3; Figure 5). Few sand flies were captured in either intervention arm at 43 days post intervention (Figure 5).

Fig. 5. Median number of *P. orientalis* captured per trap night in Experiment 2. Numbers in ODRS-treated (black lines) and control (light grey lines) households from the day of intervention, measured by sticky traps set (A) at outdor sites; (B) at peridomestic sites; and (C) by CDC light traps set at outside locations. Error bars represent the IQR (25-75 percentiles). Data collected from the 2nd May to 16th June 2017.

Table 3. Intervention (ODRS) effect estimates on *P. orientalis* sand fly catches over 43 days follow-up, by trap type and trap location.

Trap type	Trap local	IRR (95%C.I.)	P<	N trap nights
ST	O	0.04 (0.028, 0.080)	0.001	180
ST	P	0.08 (0.051, 0.113)	0.001	180
LT	O	0.01 (0.005, 0.008)	0.001	238

Trap types for monitoring: ST sticky traps, LT light trap
Trap site locations: O outdoors, P peridomestic.

Accounting for the trial design, the ODRS effect was significantly greater measured by the single LT, than by the set of 10 STs at outdoor trap locations (test of intervention × trap type interaction term: Z=2.57, P=0.010, N=418). The effect estimate was also greater measured at outdoor ST compared to at peridomestic ST locations (test of intervention × ST site-code interaction term: z=3.65, P=0.001, N=360) (Table 3).

Experiment 3

The third experiment measured the insecticide effects over 76 days follow-up of a variant of ODRS, namely where insecticide application was restricted to only household boundary fences (RODRS). Since there was no difference in effect estimates between outdoors and peridomestic ST sites under either RODRS or ODRS interventions (test of intervention × trap location: Z<0.76, P>0.393), the data for these two trap locations were combined. Across ST sites, RODRS reduced vector numbers by a mean 60% (95% CL: 19.7, 80.5%) compared to 85% (95% CL: 70.7%, 92.5%) by ODRS, both relative to in control clusters (Table 4; Figure 6). In outdoor LTs,

the equivalent estimates were 88% (95% CL: 75.7%, 94.6%) and 93% (95% CL: 84.5%, 96.6%) respectively (Table 4; Figure 6).

Table 4. Intervention (ODRS and RODRS) effect estimates on *P. orientalis* sand fly vector catches over 76 days follow-up, by trap type and trap location.

Treatment comparisons	IRR (95%C.I.)	P<	Trap type	Trap local	N trap nights[1]
ODRS vs control	0.15 (0.075, 0.293)	0.001	ST	O, P	670
RODRS vs control	0.40 (0.195, 0.803)	0.010	ST	O, P	670
ODRS vs control	0.07 (0.034, 0.155)	0.001	LT	O	349
RODRS vs control	0.12 (0.054, 0.243)	0.001	LT	O	349

Trap types and trap site location abbreviations are as shown in Table 3.

Fig. 6. Median number of *P. orientalis* captured per trap night in Experiment 3. Numbers in ODRS (black lines), RODRS (black dashed lines) and Control (light grey lines) -treated households from day of intervention, measured by sticky traps set (A) at outdoor sites; (B) at peridomestic sites; and (C) by CDC light traps set at outside locations. Error bars represent the IQR (25-75 percentiles). Data were collected from 31st March to 20th June 2017.

Comparing RODRS to ODRS intervention outcomes directly (i.e. not compared to control clusters), the RODRS effect estimates on ST catches were on average 58% (95% CL: 43.6%, 74.1%, N=446) lower than achieved by ODRS (Z= -4.09, P<0.001, N=446). In contrast RODRS

proved 100% as effective as ODRS when measured by outdoor LT captures ($Z=0.76$, $P=0.449$, $N=223$).

Economic costs of ODRS and RODRS delivery

The commercial cost of the insecticide was $15.15 USD per litre. Based on the total surface area to cover, the price per household was $3.49 USD for ODRS, and $1.89 USD for RODRS. The spray team delivered ODRS to 40 households in a single day. The joint labour costs for the three technicians was $90.91 USD per day, or $2.27 USD per household. To deliver RODRS, the labour costs fell to $1.59 USD per household. Excluding expenses of transport and administration, the relative sum costs of ODRS and RODRS delivery per household was 3.49+2.27= $5.76 USD, and 1.89+1.59= $3.48 USD, respectively. Thus, RODRS resulted in an average saving of 39.6% over ODRS.

DISCUSSION

The reported VL case incidence in East Africa (Sudan, South Sudan, Ethiopia, Somalia, Kenya, and Uganda) is now greater than that recorded in the Indian subcontinent, which was traditionally considered to suffer the heaviest VL burdens [5,30,44]. The lack of sustainable community-wide methods to reduce exophilic/exophagic disease vectors such as *P. orientalis* is a serious hindrance to efforts to reduce VL burdens in East Africa, despite recent international investment to scale-up early case detection and treatment as the only currently employed methods to combat the disease. At the time of study, the regional VL case incidence was 26–37/1000/year (2014–2017 MoH data), which is not dissimilar to published historical values of 38–39/1000/year (1991–1993) [35], 20–42/1000/year (1994–1996) [36], and 20–25/1000/year (2010–2011) [37] in the same region.

The results of the current study demonstrate that Outdoor Residual Spraying (ODRS) with pyrethroid insecticide can significantly reduce vector numbers in the outdoor compound, and in proximate peridomestic locations outside the boundary fence, where high vector numbers are known to occur [20,33]. Despite the variation in prevailing vector numbers between the two villages in this study, and the general seasonal decline in sand fly abundance, the intervention effects were consistent across experiments showing, importantly, that a single application of outdoor insecticide early in the transmission season could impact on outdoor and peridomestic sand fly vector numbers with lasting effects until the end of the transmission season, which lasts from March to June. Overall reductions of 83%-99% were achieved and remained more or less constant for the duration of the study period (maximum 76 days follow-up). The longitudinal experiments (Experiments 2 and 3) were conducted from April/May to June, representing a large fraction of the sand fly season (March to June) in east Sudan, NW Ethiopia and South Sudan. ODRS also reduced vector abundance in sleeping huts, though the numbers collected were few and insufficient to calculate robust IRR estimates.

In an attempt to reduce the quantity of insecticide and spraying effort required for ODRS, we investigated the entomological efficacy of RODRS by restricting spraying to the household boundary fence only (Experiment 3). The rationale for this alternative was based on prior observations of large numbers of vectors on the proximate exterior side of the fences. Relative to control clusters, RODRS caused an immediate suppression of outdoor and peridomestic *P. orientalis* abundance, with overall reductions during follow-up of 60% and 88% by ST and LT estimation respectively. These values compared to 85% and by 93% for ODRS in the parallel treatment arm. Direct comparisons of RODRS and ODRS effect estimates (i.e. not compared to control clusters) gave mixed results: RODRS was an average 58% as effective as ODRS based

on ST captures (P=0.001), and 100% as effective based on LT captures (P>0.05). Here, *P. orientalis* numbers captured by the two trapping methods refer to a single LT located 0.5 m above ground, as compared to an individual ST comprising 10 × A4 white paper sheets treated with sesame oil placed horizontal on the ground surface. We did not detect a consistent distinction in trap numbers (trapping efficiency) between LT and ST methods, or trapping success between outdoor *versus* peridomestic trap locations (Table S2), by which to explain the inconsistency in relative effectiveness of RODRS. Additional experiments would be useful to increase the precision of the RODRS effect estimate.

The boundary fences sprayed in this study are 1.5-2 m high constructed of tightly woven reed, which surround individual household compounds to provide privacy. The fences are likely to act as a physical barrier to blood-seeking *P. orientalis* incoming from more distant peridomestic and sylvatic habitats. Their significance as a target for ODRS/RODRS can be explained by how sand fly locomotion behaviour facilitates contact with the non-repellent pyrethroid insecticide. Sand fly vector species tend to stay close to the ground at <1 m height, and traverse the ground, flat or sloping surfaces, by a series of "hop-like" motions [45]. Observations also show that when they encounter a vertical obstacle, they proceed upwards close to the obstacle, landing on the substrate in between short flight steps [45]. Our controlled experiments indicate that vectors indeed landed on the insecticide-treated fences resulting in a lethal insecticide dose causing significant reductions in sand fly abundance. Repellence (diversion) from treated surfaces was not observed.

Such boundary fences are common across the VL endemic villages in Gedarif, Sinnar and Blue Nile states, in addition to other endemic regions of Sudan, where ODRS / RODRS could be

applied to control transmission. The findings may be relevant also to other high VL incidence regions of East Africa where *P. orientalis* is the vector, for example, amongst the vast populations of seasonal migrant agricultural workers e.g. on farms along the Sudan/Ethiopian border, where 60 % of transmission of VL in Ethiopia occurs [21,46]. These notoriously mobile populations work in the agricultural farms during two periods; May-July to prepare land for planting crops, and September-November for harvesting [46]. During the VL transmission season (May – July), the majority of workers sleep in small shelters made of grass [21]. We suggest that insecticide-impregnated fences erected around existing, or as newly built, night-time shelters could provide significant protection from the blood-seeking *P. orientalis*. In essence, insecticide sprayed fences represent sandfly barriers that can also be utilized to protect semi-nomadic populations such as cattle herders or agricultural farmers who tend to stay in temporary camping grounds.

One potential concern for the development of ODRS programmes is the expected short (e.g. 2-3 month) residuality of pyrethroids sprayed onto outdoor surfaces exposed to intense UV radiation [47]. Fortunately, the sand fly season in eastern Sudan is short (3 months) and abruptly ends with the heavy rains that occur at the end of June [17]. The heavy rains seals cracks in the soil where the vector is thought to reside [39]. One short-coming of the current study was that we did not test the residuality of the active ingredient more formally by performing sand fly exposure bioassays and/ or quantitative chemical analyses (e.g. IQK [48]and/or HPLC). Such evaluations also would be informative.

Sand fly control

At the time of writing, the IVM program in Sudan recommends IRS using pyrethroid or Bendiocarb insecticide applied twice per year (December and June), and distribution of long-lasting insecticide-impregnated bednets (LLINs, Vestergaard Frandsen, Lausanne, Switzerland) (Mr Anwar Osman Banaga Gedaref State Ministry of Health, pers. comm.). IRS and LLINs principally target endophilic vectors (modelled on endophilic malaria transmitting mosquitoes), thus precluding effectiveness against this exophilic sand fly vector. Furthermore, IRS deployment does not coincide with the season of sand fly activity. The collective studies in Sudan and NW Ethiopia lead to the consensus that *P. orientalis* has a low propensity to enter household buildings [17,21,25,26,27,28,49,50], which is corroborated by the current data showing that comparatively few *P. orientalis* were captured inside sleeping huts as measured by chemical knockdown (KD) methods. To our knowledge, there are no peer-reviewed evaluations of IRS against *P. orientalis* in East Africa. An unpublished observational study concluded that the biannual IRS campaigns conducted in Gedaref villages by the health authorities during 2008-2016 failed to have an impact on VL incidence; the authors related the findings to the outdoors biting and resting behaviour of *P. orientalis*, whereby they had limited contact with treated walls targeted by IRS [32].

Previous attempts to reduce *P. orientalis* abundance and/or *L. donovani* transmission include ultra-low volume (ULV) fogging of *A. seyal* thickets [53], provision of insect repellents [20], and ITNs [9,54,55]. Whilst the community-wide distribution of ITNs was retrospectively associated with protection against VL under epidemic conditions [9], ITNs are not generally used in resident communities during the *P. orientalis* biting season, being characterised by particularly high temperatures. During this period, people habitually sleep outside in the compound without a

bednet. ITNs tend to be used more inside huts during the rainy / cooler season (late June to October) when nuisance / malarial mosquito vectors are most abundant, but *P. orientalis* is no longer active. In a previous ITN study, which took place in Sudan, bednets were set up at 9-11pm, leaving children unprotected during a significant period of peak sand fly biting time at 6-9 pm [9]. Furthermore, the study reported bednet use by <10% of the sample population during the hot dry months (sand fly season, only rising to 55% during the beginning of the rainy season (end of sand fly season).

Intervention costs

The relative costs of RODRS and ODRS were estimated to be USD $3.48 and USD $5.76 per household, respectively, with a saving of about 40% by RODRS *versus* ODRS. These represent simple calculations for labour and insecticide, assuming similar implementation costs (transport, equipment and administration) for the two vector control strategies. A recent study in the region suggested that the cost of IRS using deltamethrin or Bendiocarb was about USD $2.2-$2.85 per person year, based on more sophisticated health economic models, concluding that IRS + LLINs were highly cost-effective for malaria control [31] as defined by WHO [56]. Direct cost comparisons with values calculated in the present study are not strictly valid, especially with variation in methods of insecticide procurement, and the rapidly declining exchange rate of the Sudanese pound. The latter may have resulted in an overestimation of the actual costs of ODRS (data not shown), nonetheless, considering 5 persons per endemic household in the region [9], ODRS and RODRS per person year would appear relatively low cost per household. Prevention is clearly less costly than VL management, with estimated direct and indirect costs of treatment for a single VL episode being $450 USD [57] The cost-benefit is likely to be even greater considering that a proportion of subsequent post-Kala azar dermal leishmaniasis (PKDL) cases

would also be avoided. Governmental and non-governmental agencies provide treatment drugs free of charge, but households are reported to bear 53% of the total costs, accounting for 40% of the median annual household income in Gedarif state [57].

In conclusion, these pilot studies establish the feasibility and potential entomological effectiveness of ODRS and RODRS as novel vector control options in Gedarif state and other endemic regions of Sudan. A key finding was that the observed levels of impact were achieved by a single application of insecticide. These community-wide interventions are likely to have high levels of social acceptability as they address the compliance issues around bednet use, and the populations are already accustomed to insecticide spraying. Although the critical threshold in sand fly abundance to achieve protection against transmission and VL is not yet known, the collective results of these pilot studies suggest that ODRS/RODRS are strong candidates for a more sustainable community-wide vector control approach in endemic regions in Sudan, and possibly elsewhere where sand fly vectors are particularly exophilic and exophagic and where building such fences may be feasible. Intervention trials to assess the impact and cost-effectiveness of this approach on *L. donovani* transmission and VL incidence are now warranted.

Supporting Information

Table S1. Total numbers of *P. orientalis* captured in the three Experiments pre- and post-intervention according to trap type and trap location.

Table S2. Median (IQR) numbers of *Phlebotomus orientalis* sandflies captured per trap night before and after outdoor residual insecticide spraying of exterior walls of sleeping huts, and household boundary fences (ODRS), or boundary fences alone (RODRS), in Gedarif state,

eastern Sudan. (a) Experiment 1 conducted in June 2016 in Jebel-Algana village; (b) Experiment 2 conducted in May-June 2017 in Umsalala village; (c) Experiment-3 conducted in March-June 2017 in Jebel-Algana village. For Experiments 2 and 3, median values were calculated over 43 and 76 days follow-up respectively.

Acknowledgements:

We would like to express our deepest gratitude to Dr. Naiema Algaseer, and other colleagues at the WHO office in Khartoum, the KalaCORE administration at Mott MacDonald (UK), the University of Khartoum, University of Gezeira, and University of Gedarif (Sudan) for facilitating the logistics and the finance of the fieldwork. Thanks are also due to several field assistants who helped in the field study. Special thanks to Mr. Hag Omer and Mr. Ahmed Hussein and the population of Belo village, Gedarif state, for hosting us throughout the project.

Funding

The authors gratefully acknowledge financial support from Department of International Development (DFID), the UK AID programme, on Tackling Visceral Leishmaniasis in East Africa, through the KalaCORE consortium (contract PO 6361) (www.kalacore.org). The funders had no role in study design, data collection and analysis, decision to publish, or preparation of the manuscript. The views expressed do not necessarily reflect the UK government's official policies.

Author contributions

Conceptualization: Dia-Eldin A Elnaiem, Orin Courtenay,

Formal analysis: Orin Courtenay, Dia-Eldin A Elnaiem

Funding acquisition: Dia-Eldin A Elnaiem, Jorge Alvar, Margriet Den Boer

Investigation: Dia-Eldin A Elnaiem, Osman Dakein, Ahmed Ali Elawad, Basheer M Basheer, Eltayieb Khogali, Noteila Khalid, Taiseer Gibreel, Omran F Osman, Hassan Has'san

Methodology: Dia-Eldin A Elnaiem, Orin Courtenay,

Project administration: Dia-Eldin A Elnaiem, Mousab S Elhag, Atia Abdulla, Omran F Osman

Resources: Dia-Eldin A Elnaiem, Orin Courtenay

Supervision: Dia-Eldin A Elnaiem.

Data analysis: Orin Courtenay, Dia-Eldin A Elnaiem

Writing – original draft: Dia-Eldin A Elnaiem, Orin Courtenay,

Writing – review & editing: Orin Courtenay, Dia-Eldin A Elnaiem, Jorge Alvar, Margriet Den Boer, Caryn Bern, Koert Ritmeijer, Noteila Khalid.

References.

1. World Health Organization (WHO). Control of the leishmaniasis. Report of meeting of the WHO expert committee on the control of leishmaniasis. World Health Organization, Geneva, technical report series NO 949, 2010.
2. World Health Organization (WHO). Accelerating work to overcome the global impact of neglected tropical diseases: a roadmap for implementation: executive summary, 2012. https.int://apps.who /iris/handle/10665/70809?locale=ar.
3. Molyneux DH, Savioli L, Engels D. Neglected tropical diseases: progress towards addressing the chronic pandemic. The Lancet, 2016. doi: 10.1016/S0140-6736(16)30171-4.
4. Alvar J, Vélez ID, Bern C, Herrero M, Desjeux P, et al. Leishmaniasis worldwide and global estimates of its incidence. PLoS ONE. 2012;7:e35671.

5. World Health Organization. Weekly epidemiological record. 2016; (accessed 29/9/19). https://wwwwhoint/wer/2016/wer9122pdf?ua=1 91:285-296.

6. Seaman J, Mercer AJ, Sondorp E. The epidemic of visceral leishmaniasisin Western Upper Nile, southern Sudan: course and impact from 1984 to 1994. Int Epidemiol. 1996;25:862-871.

7. Zijlsta EE, el-Hassan AM. Leishmaniasis in Sudan. visceral leishmaniasis.Trans R Soc Trop Med Hyg. 2001; 95:S27-S58.

8. Zijlstra EE, El-Hassan AM. Post kala-azar dermal leishmaniasis. Trans R Soc Trop Med Hyg. 2001; 95:S59-S76.

9. Ritmeijer K, Davies C, Zorge R, Wang SJ, Schorscher J, et al. Evaluation of a mass distribution programme for fine-mesh impregnated bednets against visceral leishmaniasis in eastern Sudan. Trop Med Intern Health. 2007;12:404-414.

10. Herrero M, Orfanos G, Argaw D, Mulugeta A, Aparicio P, et al. Natural history of a visceral leishmaniasis outbreak in highland Ethiopia. The Amer J Trop Med & Hyg. 2009;81:373-377.

11. Abubakar A, Ruiz-Postigo JA, Pita J, Lado M, Ben-Ismail R, et al. Visceral leishmaniasis outbreak in South Sudan 2009–2012: epidemiological assessment and impact of a multisectoral response. PLoS Negl Trop Dis. 2014;8:(3):e2720.

12. Adam GK, Ali KM, Abdella YH, Omar SM, Ahmed MAA, et al. Trend in cumulative cases and mortality rate among visceral leishmaniasis patients in eastern Sudan: a 14-year registry, 2002–2015. Inter J Infect Dis. 2016;51: 81-84.

13. Sunyoto T, Adam GK, Atia AM, Hamid Y, Babiker RA, et al. “Kala-azar is a dishonest disease”: community perspectives on access barriers to visceral leishmaniasis (kala-azar) diagnosis and care in southern Gadarif, Sudan. Am J Trop Med Hyg. 2018;98:1091-1101.

14. Sunyoto T, Boelaert M, Meheus F. Understanding the economic impact of leishmaniasis on households in endemic countries: a systematic review. Expert Rev Anti-infect Ther. 2019;17: 57-69.

15. Quate LW. Leishmaniasis in the Sudan Republic 19. phlebotomus sandflies of the Paloich area in the Sudan (Diptera: Psychodidae). J Med Entomol. 1964;1:213-268.

16. Hoogstraal H, Heyneman D. Leishmaniasis in the Sudan Republic.30. Final epidemiological report. Am J Trop Med Hyg. 1969;18:1091-1210.

17. Elnaiem DA, Hassan HK, Ward RD. Phlebotomine sandflies in a focus of visceral leishmaniasis in a border area of eastern Sudan. Ann Trop Med Parasitol. 1997;91:307-318.

18. Elnaiem DA, Connor SJ, Thomson MC, Hassan MM, Hassan HK, et al. Environmental determinants of the distribution of *Phlebotomus orientalis* in Sudan. Ann Trop Med & Paras. 1998;92:877- 887.

19. Elnaiem DA, Hassan HK, Ward RD (1999) Associations of *Phlebotomus orientalis* and other sandflies with vegetation types in the eastern Sudan focus of kala-azar. Med Vet Entomol. 1999;13:198-203.

20. Elnaiem DA. Ecology and control of the sand fly vectors of *Leishmania donovani* in East Africa, with special emphasis on *Phlebotomus orientalis*. J Vector Ecol. 2011;36: S23-S31.

21. Lemma W, Tekie H, Balkew M, Gebre-Michael T, Warburg A, et al. Population dynamics and habitat preferences of *Phlebotomus orientalis* in extra-domestic habitats of Kafta Humera lowlands--kala azar endemic areas in Northwest Ethiopia. Parasit Vectors. 2014;7:359.

22. Elnaiem DA, Osman OF. Evidence for active transmission of visceral leishmaniasis within a village in eastern Sudan. Acta Trop. 1998;71:305-309.

23. Elnaiem DA, Hassan MM, Maingon R, Nureldin GH, Mekawi AM, et al. The Egyptian mongoose, *Herpestes ichneumon*, is a possible reservoir host of visceral leishmaniasis in eastern Sudan. Parasitol. 2001;122:531-536.
24. Hassan MM, Elraba'a FM, Ward RD, Maingon RDC, Elnaiem DA. Detection of high rates of in-village transmission of *Leishmania donovani* in eastern Sudan. Acta Trop. 2004;92:77-82.
25. Gebresilassie A, Kirstein OD, Yared S, Aklilu E, Moncaz A, et al. Species composition of phlebotomine sand flies and bionomics of *Phlebotomus orientalis* (Diptera: Psychodidae) in an endemic focus of visceral leishmaniasis in Tahtay Adiyabo district, northern Ethiopia. Parasit Vectors. 2015;8:248.
26. Yared S, Gebresilassie A, Akililu E, Balkew M, Warburg A, et al. Habitat preference and seasonal dynamics of *Phlebotomus orientalis* in urban and semi-urban areas of kala-azar endemic district of Kafta Humera, northwest Ethiopia. Acta Trop. 2017;166: 25-34.
27. Aklilu E, Gebresilassie A, Yared S, Kindu M, Tekie H, et al. Studies on sand fly fauna and ecological analysis of *Phlebotomus orientalis* in the highland and lowland foci of kala-azar in northwestern Ethiopia. PLoS ONE. 2017;12: e0175308.
28. Kirstein OD, Skrip L, Abassi I, Iungman T, Horwitz BZ, et al. A fine scale eco-epidemiological study on endemic visceral leishmaniasis in north ethiopian villages. Acta Trop. 2018;183: 64-77.
29. WHO (2017) Global vector control response 2017–2030. Geneva: Contract No.: CC BY-NC-SA 3.0 IGO. https://www.who.int/vector-control/publications/global-control-responses/en/.
30. World Health Organization (WHO). Leishmaniasis. Sudan. 2018 (accessed 28/9/2019). https://wwwwhoint/leishmaniasis/burden/Sudan_2015_updatedpdf?ua=1.

31. Kafy HT, Ismail BA, Mnzava AP, Lines J, Abdin MSE, et al. Impact of insecticide resistance in *Anopheles arabiensis* on malaria incidence and prevalence in Sudan and the costs of mitigation. Proc Nat Acad Sci. 2017;114:E11267-E11275.

32. Alawad AE. Evaluation of the impact of spraying of residual insecticides on *Phlebotomus orientalis* sandflies and incidence of kala azar in Gedarif state. MSc. theis, University of Geziera, Geziera- Sudan 2017.

33. Elnaiem DA, Khogali AA, Gibreel T, Elawad AM, Dakein OM, et al. Novel approaches for control of *Phlebotomus orientalis*, the principal vector of visceral leishmaniasis in East Africa. In: worldleish2017. Abstract Book-W-6- 2017. pp. 60.

34. Elnaiem DA, Judith Schorscher, Anna Bendall, Valerie Obsomer, Maha E. Osman, et al. Risk mapping of visceral leishmaniasis: the role of local variation in rainfall and altitude on the presence and incidence of kala-azar in eastern Sudan. Am J Trop Med Hyg. 2003;68: 10–17.

35. Zijlstra EE, El-Hassan AM, Ismael A, Ghalib HW. Endemic kala-azar in eastern Sudan: a longitudinal study on the incidence of clinical and subclinical infection and post-kala-azar dermal leishmaniasis. Am J Trop Med Hyg. 1994;51: 826-836.

36. Khalil EAG, Zijlstra EE, Kager PA, El Hassan AM. Epidemiology and clinical manifestations of *Leishmania donovani* infection in two villages in an endemic area in eastern Sudan. Trop Med Intern Heal. 2002;7: 35-44.

37. Mueller YK, Nackers F, Ahmed KA, Boelaert M, Djoumessi J-C, et al. Burden of visceral leishmaniasis in villages of eastern Gedaref state, Sudan: an exhaustive cross-sectional survey. PLoS Negl Trop Dis. 2012;6: e1872.

38. Nackers F, Mueller YK, Salih N, Elhag MS, Elbadawi ME, et al. Determinants of visceral leishmaniasis: a case-control study in Gedaref state, Sudan. PLoS Negl Trop Dis. 2015;9: e0004187.

39. Moncaz A, Kirstein O, Gebresillassie A, Lemma W, Yared S, et al. Characterization of breeding sites of *Phlebotomus orientalis*-The vector of visceral leishmaniasis in northwestern Ethiopia. Acta Trop. 2014;139:5-14.

40. Morrison AC, Ferro C, Pardo R, Torres M, Wilson ML, et al. Nocturnal activity patterns of *Lutzomyia longipalpis* (Diptera: Psychodidae) at an endemic focus of visceral leishmaniasis in Colombia. J Med Entomol. 1995;32: 605-617.

41. Dye C. The logic of visceral leishmaniasis control. Am J Trop Med Hyg 55: 125 - 130.

42. Galvis-Ovallos F, Casanova C, Pimentel Bergamaschi D, Bianchi Galati EA (2018) A field study of the survival and dispersal pattern of *Lutzomyia longipalpis* in an endemic area of visceral leishmaniasis in Brazil. PLoS Negl Trop Dis. 1996;12: e0006333.

43. Kirk RO, Lewis DJ. The Phlebotominae of the Ethiopian Region. Trans Roy Soc Trop Med Hyg. 1951;102: 383-509.

44. World Heath Organization (WHO). Leishmaniasis. India. 2018; (accessed 28/9/19). https://wwwwhoint/leishmaniasis/burden/India_2015-hlpdf?ua=1.

45. Faiman R, Kirstein O, Moncaz A, Guetta H, Warburg A. Studies on the flight patterns of foraging sand flies. Acta Trop. 2011;120: 110-114.

46. Alemayehu M, Paintain L, Adera C, Berhe R, Gebeyehu A, et al. Impact of education on knowledge and practice of kala azar preventive measures among seasonal and migrant agricultural workers in northwest Ethiopia. Am J Trop Med Hyg. 2020;102: 758-767.

47. Liu P, Liu Y, Liu Q, Liu J. Photodegradation mechanism of deltamethrin and fenvalerate. J Environ Sci (China). 2010;22: 1123-1128.

48. Russell TL, Morgan JC, Ismail H, Kaur H, Eggelte T, et al. Evaluating the feasibility of using insecticide quantification kits (IQK) for estimating cyanopyrethroid levels for indoor residual spraying in Vanuatu. Malar J. 2014;13: 178.

49. Lambert M, Dereure J, El-Safi SH, Bucheton B, Dessein A, et al. The sandfly fauna in the visceral-leishmaniasis focus of Gedaref, in the Atbara-River area of eastern Sudan. Ann Trop Med Parasitol. 2002;96: 631-636.

50. Mohammed MA, Khalid NM, Aboud MA. Kala-azar in Darfur: evidence for indigenous transmission in Al-Malha Locality, North Darfur, western Sudan. Parasit Vectors. 2018;11:149.

51. Hassan MM, Widaa SO, Osman OM, Numiary MSM, Ibrahim MA, et al. Insecticide resistance in the sand fly, *Phlebotomus papatasi* from Khartoum State, Sudan. Parasit Vectors. 2012;5(1): 46.

52. Coleman M, Foster GM, Deb R, Pratap Singh R, Ismail HM, et al. DDT-based indoor residual spraying suboptimal for visceral leishmaniasis elimination in India. Pro Nat Acad Sci. 2015;112: 8573-8578.

53. Türner ER, Labrecque GC, Hoogstraal H. Leishmaniasis in the Sudan Republic. 24. Effectiveness of insecticides as residues and fogs for control of *Phlebotomus* langeroni *orientalis* Parrot (Diptera : Psychodidae). J Egypt Public Heal Assoc. 1956;40:59-64.

54. Elnaiem DA. Use of pyrethroid impregnated bednets for control of visceral leishmaniasis in eastern Sudan. Final Report (WHO ID L3/1881/47) WHO, 1996.

55. Elnaiem DA, Elnahas AM, Aboud MA. Protective efficacy of lambdacyhalothrin-impregnated bednets against *Phlebotomus orientalis*, the vector of visceral leishmaniasis in Sudan. Med Vet Entomol. 1999;13(2):191-197.

56. World Health Organization (WHO). Making Choices in Health: WHO Guide to Cost-Effectiveness Analysis. 2003. https://www.who.int/choice/book/en/.

57. Meheus F, Abuzaid AA, Baltussen R, Younis BM, Balasegaram M, et al. The economic burden of visceral leishmaniasis in Sudan: an assessment of provider and household costs. Am J Trop Med Hyg. 2013;89. Doi:10.4269/ajtmh.12-0585.

Figure 1

Click here to download Figure Fig 1.tiff

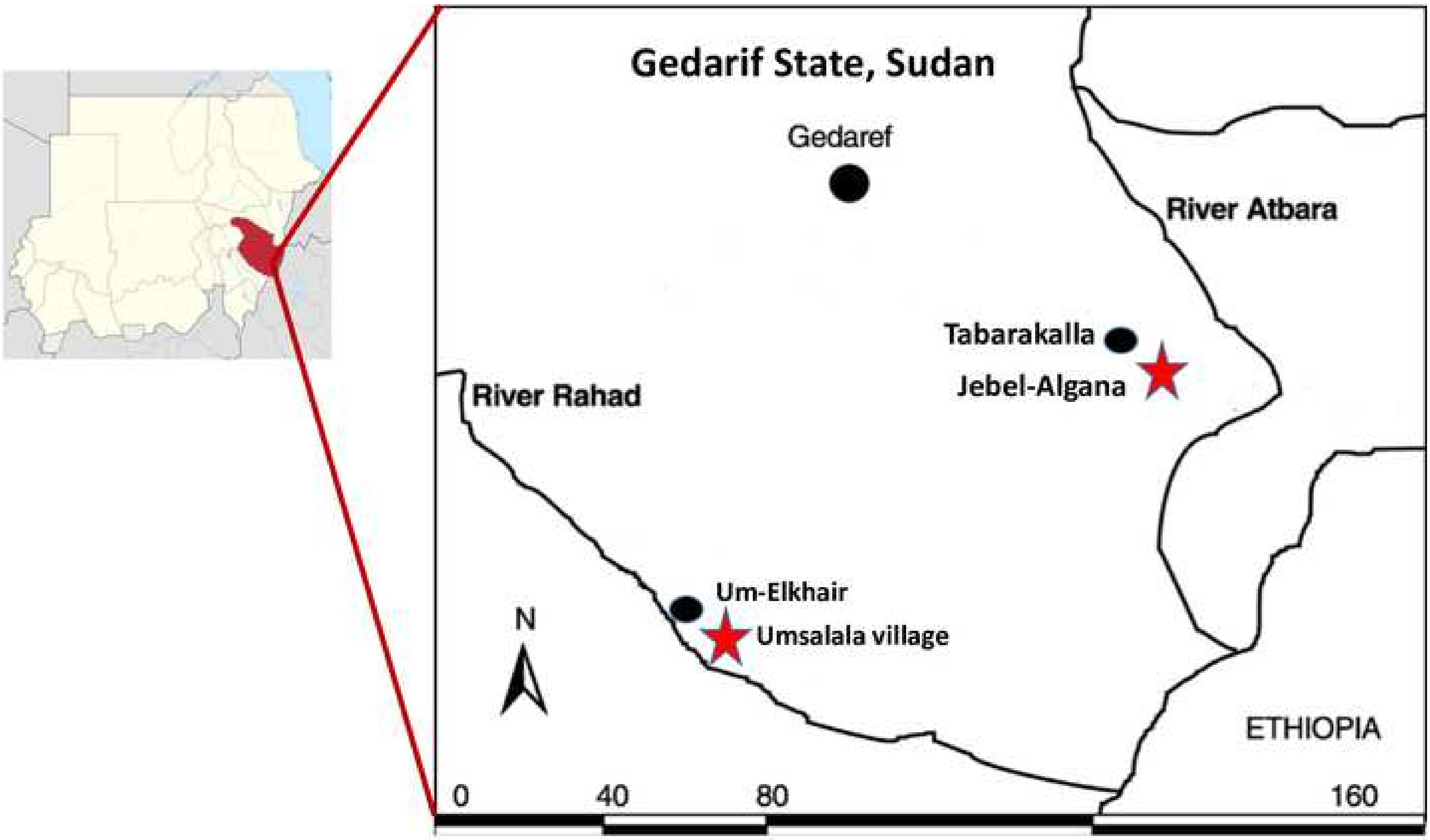

Fig.1:

 Click here to download Figure Fig 2.tiff

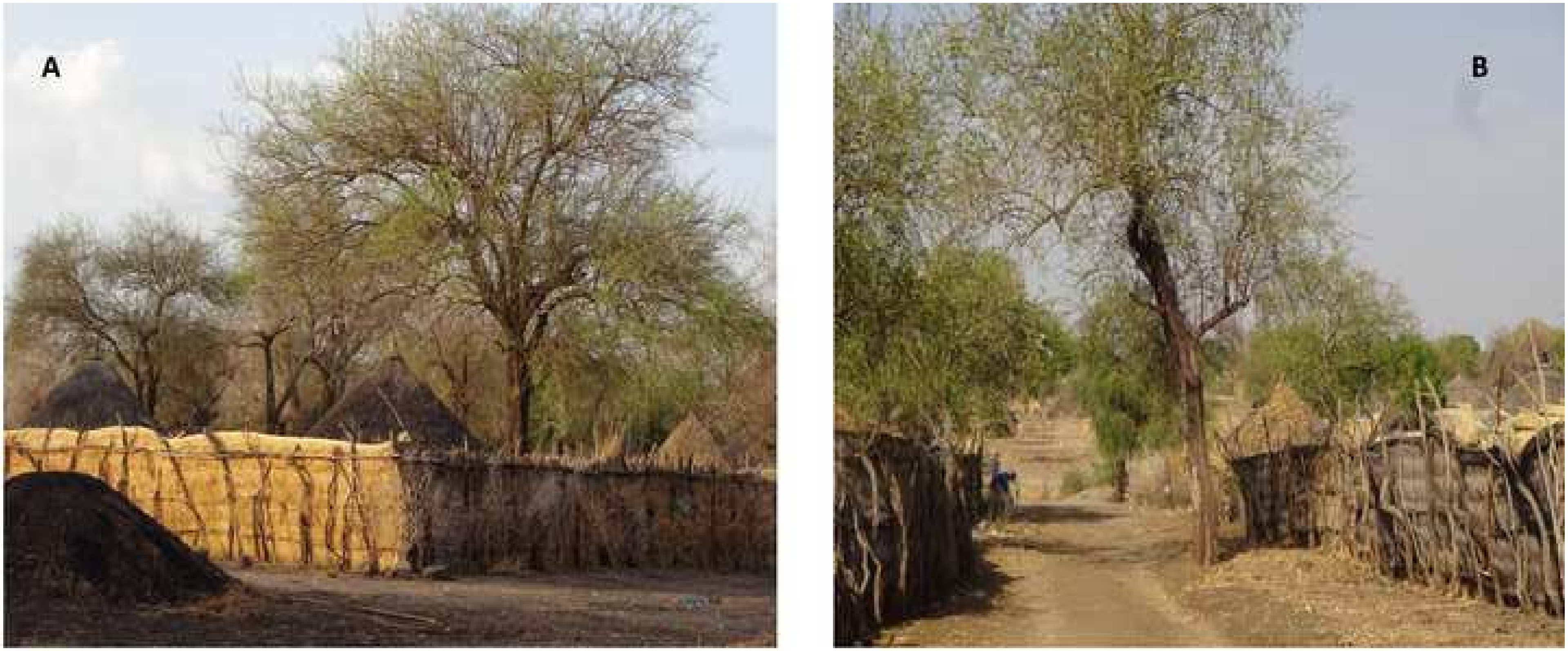

Fig.2

Figure 3

Click here to download Figure Fig 3 .tiff

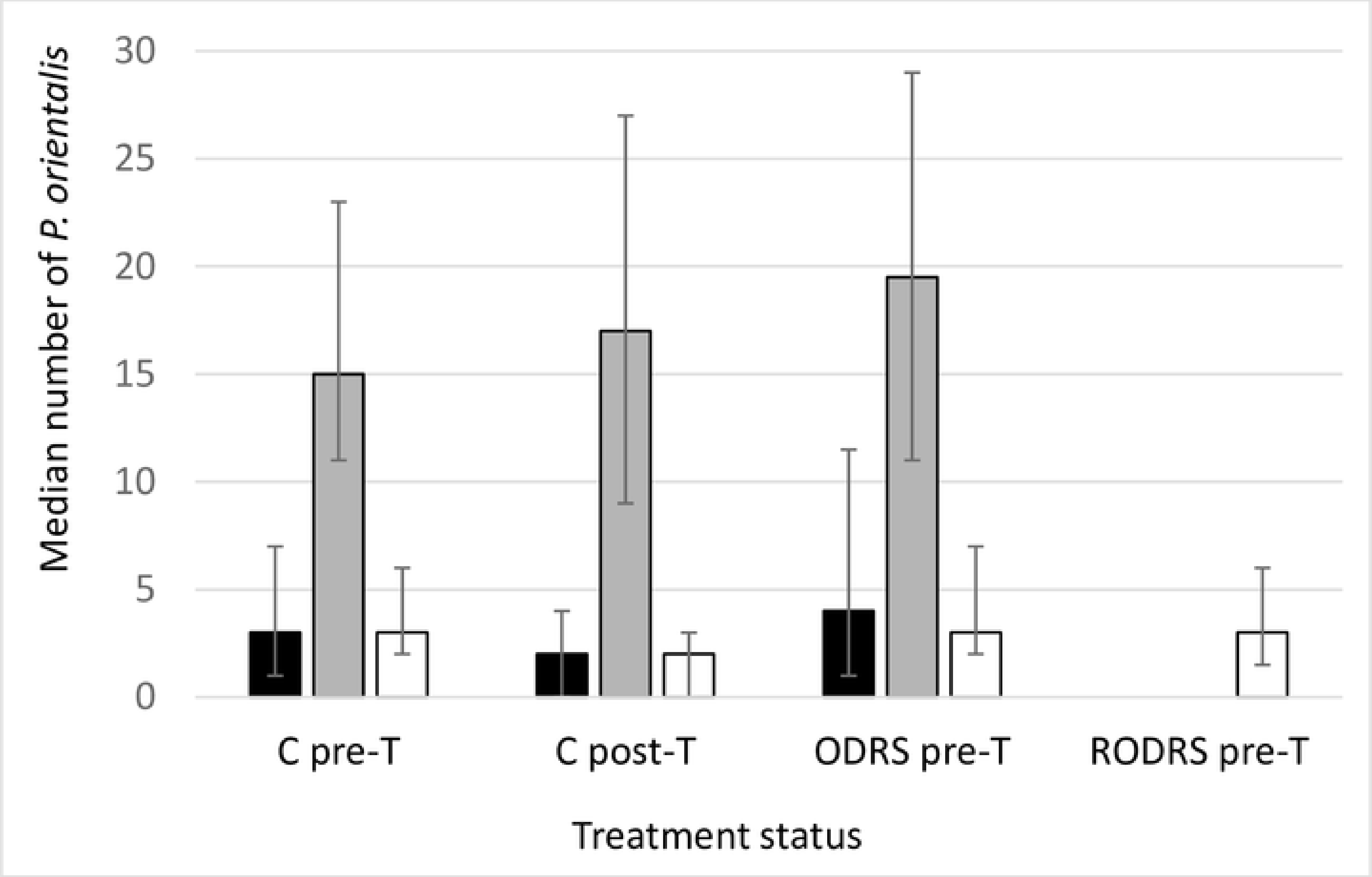

Figure 4 Click here to download Figure Fig 4.tiff

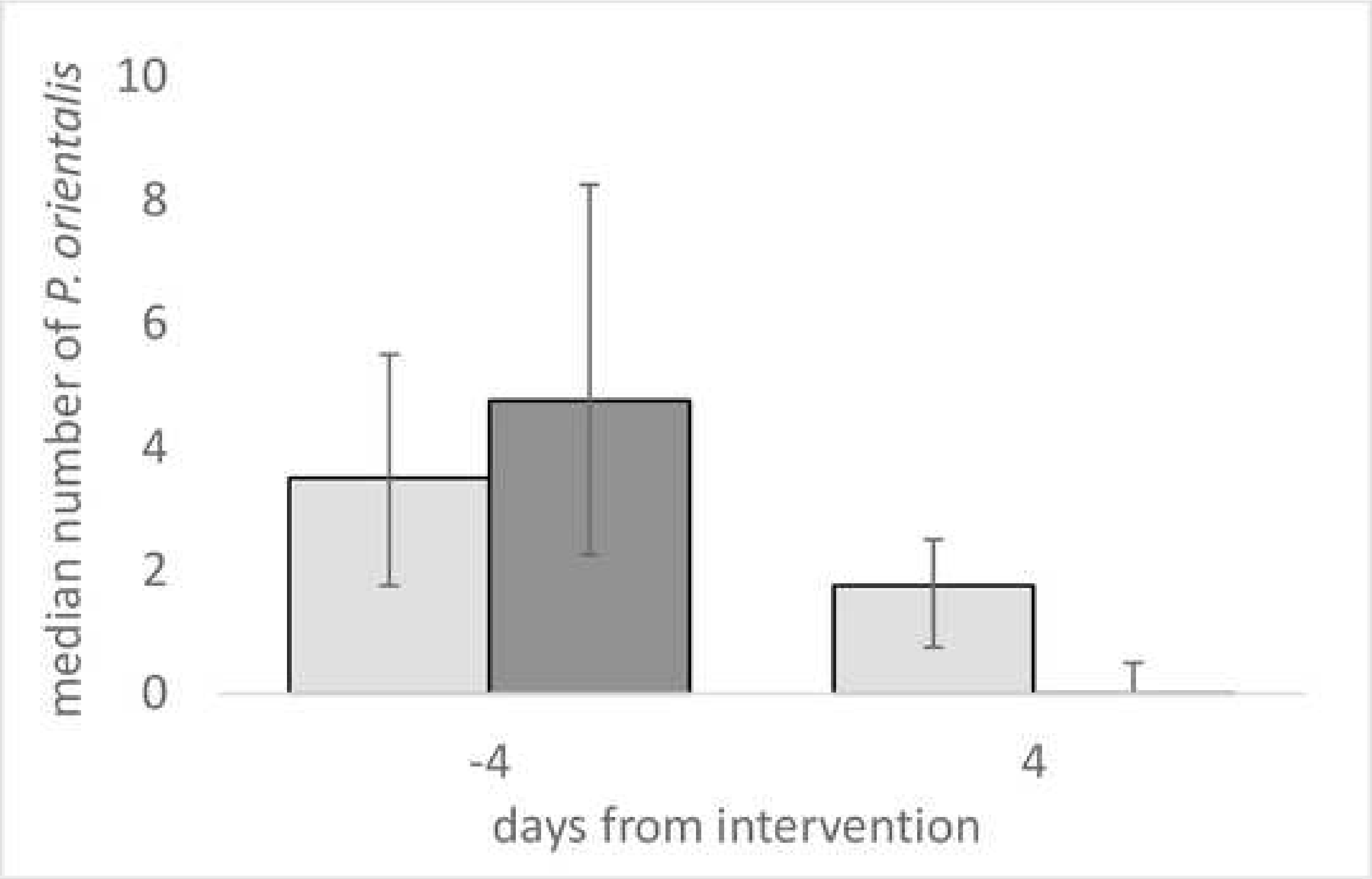

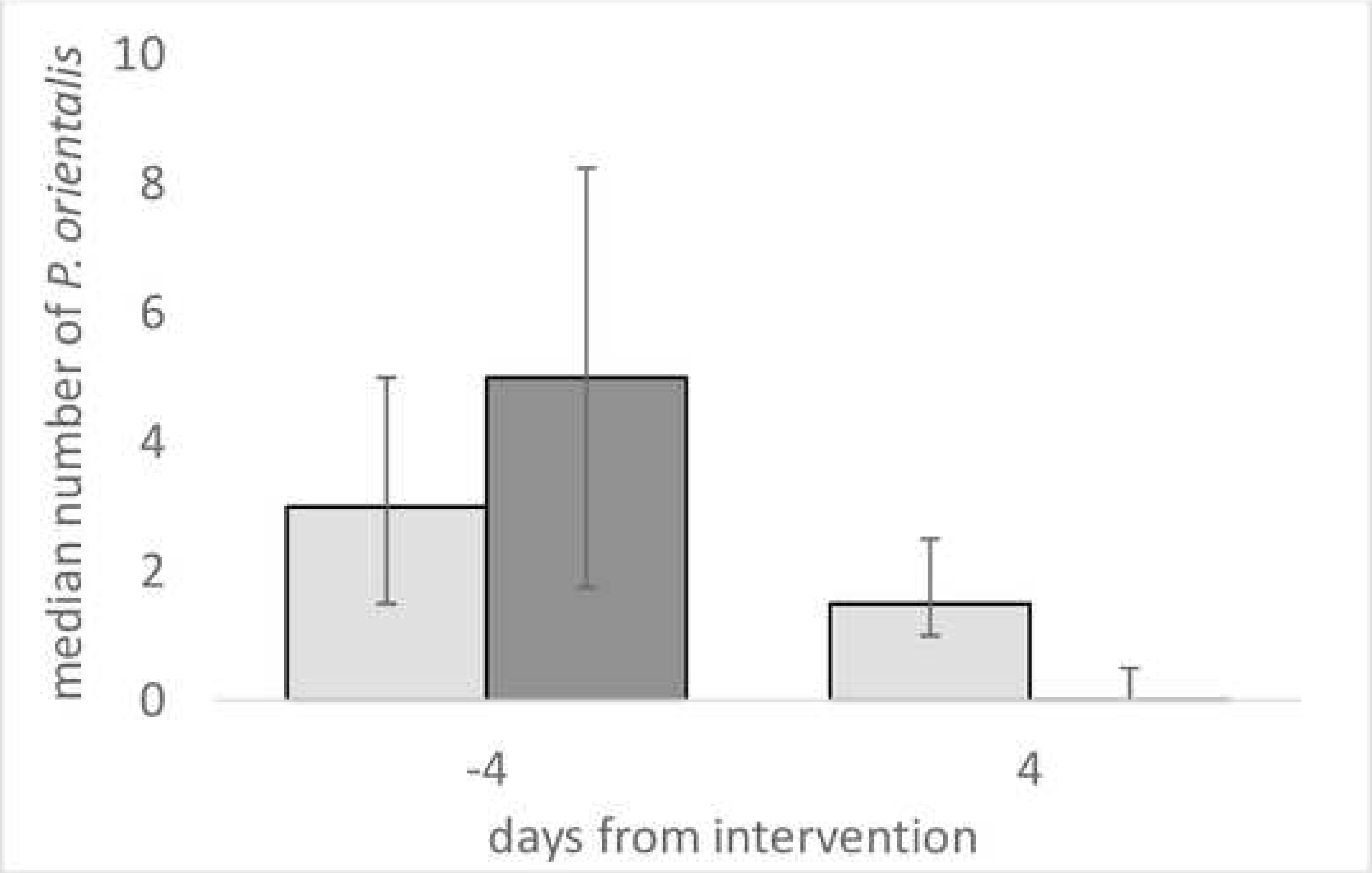

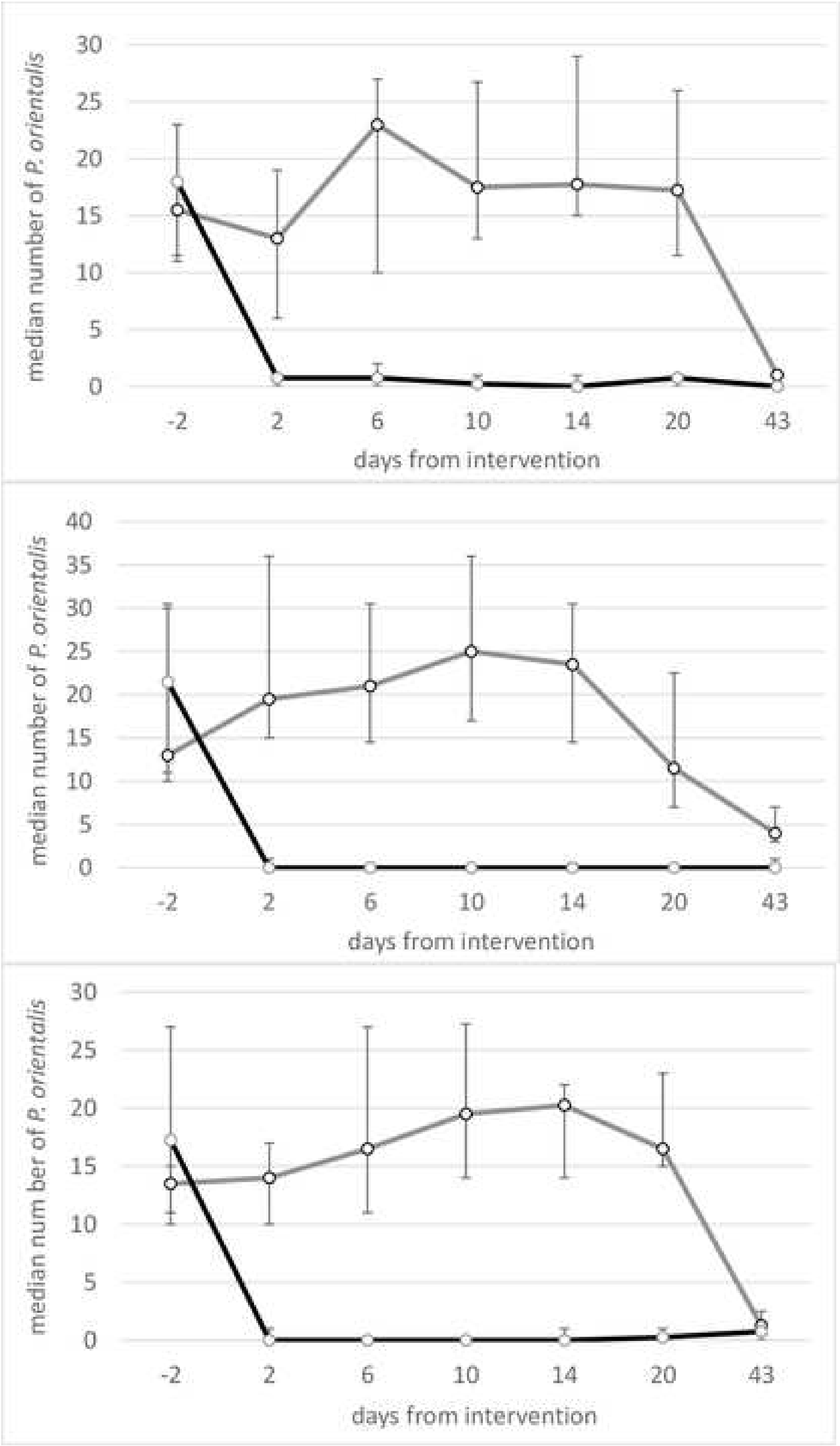
median number of P. orientalis
30
25
20
15
10
5
0
-2
2
6
10
14
20
43
days from intervention
median number of P. orientalis
40
35
30
25
20
15
10
5
0
-2
2
6
10
14
20
43
days from intervention
median num ber of P. orientalis
30
25
20
15
10
5
0
-2
2
6
10
14
20
43
days from intervention

Figure 6

Click here to download Figure Fig 6 .tiff

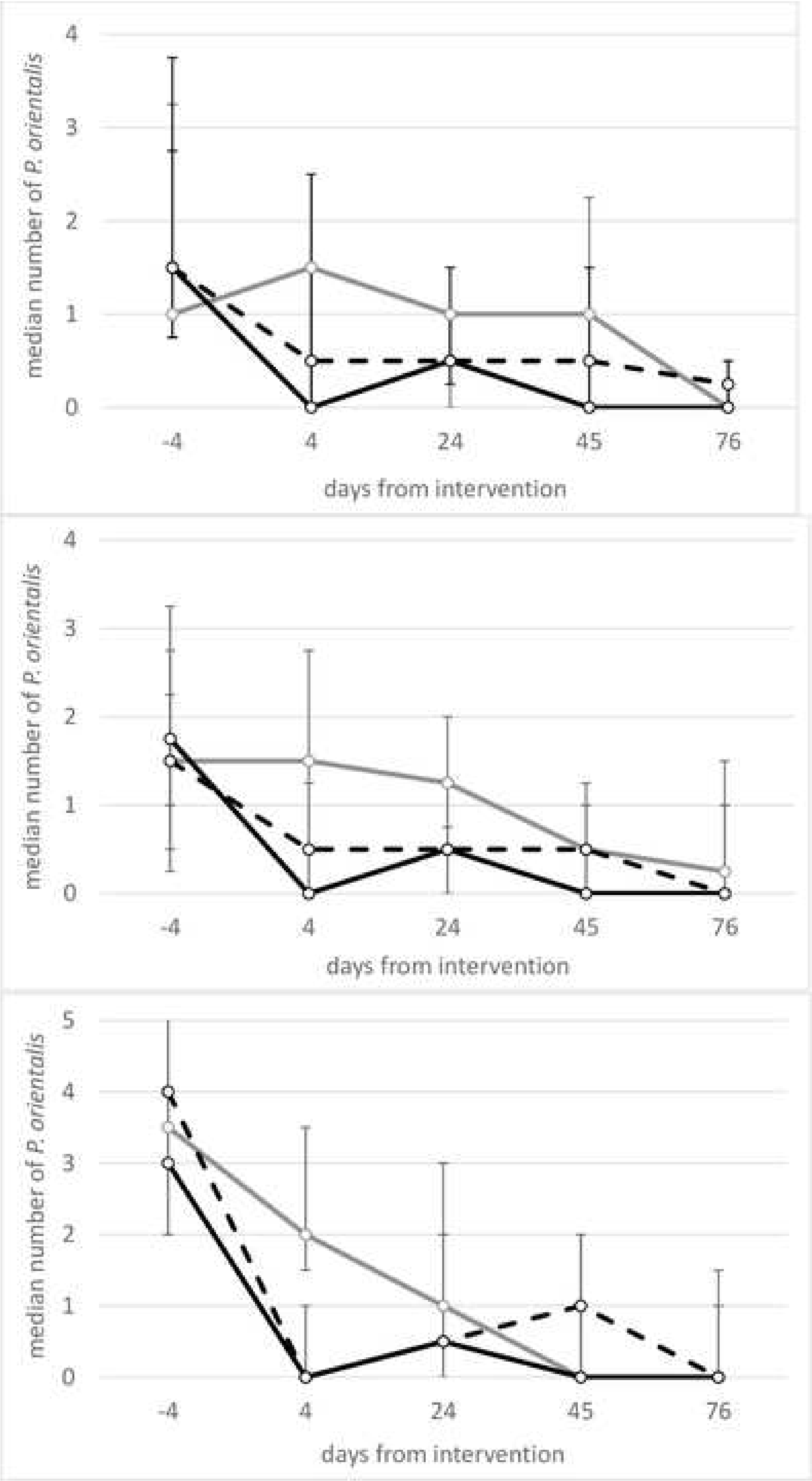

Supporting Information Table 1

Click here to access/download

Supporting Information

ODRS-Table S1-abc-Jun-4-2020.docx

Supporting Information Table 2

Click here to access/download
Supporting Information
ODRS-Table S2a-c-Jun-4-2020.docx
