# Supplementary material for: Outdoor Residual Insecticide Spraying (ODRS), a New Approach for the Control of the Exophilic Vectors of Human Visceral Leishmaniasis: Phlebotomus orientalis in East Africa
Source: PLoS Negl Trop Dis. 2020 Oct 20;14(10):e0008774. doi: 10.1371/journal.pntd.0008774 (PMC7598920; doi:10.1371/journal.pntd.0008774)
Supplement: Attachment — Submitted filename: Elnaiem-ODRS-Response-to reviewers-comments-Aug-28-2020 .pdf [file pntd.0008774.s005.pdf]

UNIVERSITY of MARYLAND
EASTERN SHORE

SCHOOL of AGRICULTURAL AND NATURAL SCIENCES
Department of Natural Sciences

Aug 28, 2020

Joachim Clos
Associate Editor
PLOS Neglected Tropical Diseases
and
Shan Lv
Deputy Editor
PLOS Neglected Tropical Diseases
Dear Editor

Re: (PNTD-D-20-00991) - [EMID: fa2b72040159e68b]. Response to the review of our manuscript "Outdoor residual insecticide spraying, a new approach for the control of the exophilic vector of human visceral leishmaniasis: *Phlebotomus orientalis* in East Africa".

Dear editors,

Thank you for the careful consideration of the above-mentioned manuscript for publication in "PLoS Neglected Tropical Diseases". We very much appreciate the helpful comments received from the reviewers, which have improved the write-up of the paper.

Our point-by-point response to each comment and suggested correction made by the reviewers is given below and also highlighted in the revised text. In addition to the reviewers suggested changes, we made few minor corrections, which are also highlighted in green color and listed at the end of this letter.

Response to comments in the letter from the editor:

The manuscript entitled "Outdoor residual insecticide spraying, a new approach for the control of the exophilic vectors of human visceral leishmaniasis: Phlebotomus orientalis in East Africa" describes an interesting study. The results of this study are of major eco-epidemiological importance and should lead to a better control of visceral leishmaniasis in East Africa. The manuscript should be published after taking into consideration the following comments:

Introduction
The authors should report the curve showing the phenology of P. orientalis in Soudan.

>>In this study, we did not measure the seasonality of the vector. However, in response to the reviewer suggestion, we have added the documented literature information on the phenology of *P. orientalis* and the seasonality of transmission of visceral leishmaniasis in the area (see the end of the third paragraph of the Introduction section).

Carver Hall, Room 1103 Princess Anne, Maryland 21853 Tel: (410) 651-6013/6015 Fax: (410) 651-7739

Methods

The authors should use the sticky traps similarly to light traps placed 1 m above the ground and not placed on the ground.

>>sticky traps were placed on the ground as our recent work confirmed previous reports that sticky traps are more effective in capturing *P. orientalis* when placed on the ground compared to a vertical position. Actually, the horizontal position result in an 8-fold increase in the number of *P. orientalis* captured per trap night. Reference to this recent published work has been added to the Methods (under Sand fly sampling) and Discussion (7th paragraph) of the manuscript.

ST are not equivalent to CDC light traps as they are inherently different trapping mechanisms. We acknowledge this by analysing the intervention outcomes separately for the two trapping methods, and all results pre and post intervention are comparative in any case.

ST traps should be used to report the densities of P. orientalis (number of sandflies per m² of ST).

>>We report numbers of vectors per sampling unit (single CDC light trap or set of 10 ST papers [10 x A4 sheets]) consistently throughout the text. The ST sampling unit could of course be converted to numbers per m^2; however, we feel that it is confusing to report numbers per m^2 for ST but not for CDC light traps. We are aware that some authors report density of sandflies per $m^{2;}$ however, this is not universal and has shortcomings. We intentionally present our data as actual numbers, avoiding extrapolation as the numbers do not necessarily reflect vector abundance. Again, the numbers are used comparatively in the intervention analyses. However, in response to the reviewer's request, we have added a comment to the Discussion considering the total ST paper area and the implied fly densities per m^2 (see end of paragraph 7 of the Discussion section).

The authors should use the Abott's formula to report the reduction in the abundance of sandflies.

>> The statistical methods employed to estimate changes in the abundance of sand flies use multivariate negative binomial models that include model offset, cluster term, and quadratic term to test for non-linearities in the data. The effect estimates are expressed as incidence risk ratio (IRR) which as defined in Methods is the response relative to the control (background), whilst accounting for starting conditions, trial design, allowing for variation between intercepts and slopes. Abott's formula is a simplistic estimation correction that fails to account for the study design features, clustering, etc, and does not even provide a reliable measure of variance. Hence, we strongly disagree with the referee's request and prefer to maintain the more appropriate method of analysis as reported.

In addition to reduction of sandfly's abundance, the author should report the infection rate of P. orientalis with L. donovani.

>>this was beyond the scope of the study and therefore was not tested. Indeed, in future studies it would be interesting to measure this variable along with the impact on disease incidence. We have added this comment to the discussion (see last sentence of the Discussion section).

Conclusion

While IRS and ITN showed their limitations, this approach has a great potential to be used in controlling VL in East Africa where the number of cases exceeded the Indian subcontinent.

Carver Hall, Room 1103 Princess Anne, Maryland 21853 Tel: (410) 651-6013/6015 Fax: (410) 651-7739

Additional comments noted on the manuscript

Abstract and Author's summary
>>Alterations to the Abstract and Author's summary have been made for clarity as suggested by the referee.

>>In the abstract we provided the full names for the acronym ODRS as suggested. We also edited the last sentence in the first paragraph to read "The effects of intervention on sand fly numbers".

Authors summary

>>We provided the full name before the ACRONYM as suggested by reviewers

>>We wrote Restricted Outdoor Spraying, as suggested by the reviewer

Introduction

Second paragraph:

>>We mean highest incidence worldwide as written. Please, note the minor correction made in the sentence to make it clearer.

Last paragraph:
The referee questions the use of the term "entomological efficacy"
>>"entomological efficacy" is used in the manuscript to differentiate it from its usual clinical definition, which is to reduce human or animal infection rates. Hence, we prefer to keep this term.

Methods
Second Paragraph:
The referee requests the long sentence be split for clarity
>>the sentence has been altered to read: "The stability in *P. orientalis* biting rates in the region is indicated by the consistent human VL incidence rates in previous and current years". The supporting data for this statement is already provided in the Discussion.

Third Paragraph:
>>We have corrected the word "*Balanites*".

First paragraph under the subtitle Interventions:
The referee questions if the insecticide formulation is micro-encapsulated.
>>The formulation is already described in the text. It is clear from the standard signature in the formulation name "WP" (wettable powder) that it is not a micro-encapsulated formulation which would be "ME".

Results

Table 1 + others

Please provide detailed legend to table. Contents- Are the numbers given the "mean No. of sand flies per trap per night? Seems too high!? If not, what are the numbers totals, how many trap/nights? same number of trap nights for each one? same traps used for calculating all "trap nights".

>>The Table legends have been altered to describe the contents of the Tables more clearly. The numbers and totals in Table 1 have been checked carefully. They correspond to the totals cited in the text.

Figures

We edited all figures and figure legends as requested by the reviewers.

>>Fig 3. As suggested by the referee we have added the number of trap nights to the figure legend as explained for Fig 4-6 below.

Label and explain the panels of the figures. These figures (Fig 4-6) are somewhat misleading because the X axis intervals are unequal lengths rendering the slopes of the curves not meaningful. Please consider bar graphs instead. Address comments (where relevant) in other tables as well as this one.

>>Labels A and B, or A, B, C have been added to the panels in Figures 4-6.
We agree with the referee's suggestion to provide numbers of trap nights per experiment to the Figures. However, to avoid cluttering the Figures, we have stated sample sizes in the Figure legends, and have revised the legends to explain more clearly the contents of each panel.
The x-axes have been altered in Figures 4-6 to represent the actual time intervals between sampling points.

Discussion

First Paragraph:

The referee requests the long sentence be split for clarity

>> the sentence has been altered to read: "The lack of sustainable methods to control exophilic/exophagic disease vectors such as *P. orientalis* is a serious hinderance to efforts to reduce VL burdens in East Africa. This is despite recent international investment to scale-up early case detection and treatment as the only currently employed methods to combat the disease".

Comment for discussion

What is the reason for the sharp decline in numbers in control traps between days 20 and 43? If its end of season, this also affected your experimental traps preventing resurgence of numbers due to solar inactivation of your insecticide.

>> Yes, the end of the season is responsible for the sharp declines in sand fly numbers which coincides with the onset of the seasonal rains. Therefore, solar inactivation of insecticides should not compromise the efficacy of this method. We already have a paragraph in the Discussion stating the advantage of the short season of the vector in strengthening the outcome of this control measure. To this paragraph, we also added a sentence explaining that the fall in the vector abundance between day 20 and 43 in experiment 2 and days 45-76 in experiment 3 were associated with the beginning of the rains (see Paragraph 7 of Discussion).

How come decline in sand fly numbers depicted in figure 6, was a lot longer? 45-76 days?

>> Figure 6 shows results from experiment 3 which was conducted over a longer follow-up period than experiment 2, as it was started about a month earlier than experiment 2, hence the last sample was on day 76. All treatment outcomes are comparative to control clusters in any case.

Additional Corrections made by the authors:

1. We corrected the spelling of the names of three of the co-authors. These corrected spellings have already been suggested by the co-authors themselves and conveyed to the journal.
2. We deleted two references (reference number 51 and 52 in the original document), which were not cited in the text.
3. To substantiate the statements made in response to the reviewers we added 4 new references (number 29, 30, 45 and 48 in the revised document).
4. The numbers and orders of references from 29 to the end has been changed to accommodate above changes.

Thank you for your consideration of publication of this manuscript.

Sincerely,

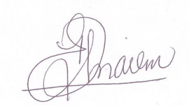

Dia-Eldin A. Elnaiem
Professor of Applied Zoology, Department of Natural Sciences, University of Maryland Eastern Shore, 1 Backbone Road, Princess Anne, MD 21853,Tel: 410-651-6031; Email: daelnaiem@umes.edu
