# Supplementary material for: Outdoor Residual Insecticide Spraying (ODRS), a New Approach for the Control of the Exophilic Vectors of Human Visceral Leishmaniasis: Phlebotomus orientalis in East Africa
Source: PLoS Negl Trop Dis. 2020 Oct 20;14(10):e0008774. doi: 10.1371/journal.pntd.0008774 (PMC7598920; doi:10.1371/journal.pntd.0008774)
Supplement: Attachment — Submitted filename: Comments.doc [file pntd.0008774.s004.doc]

The manuscript entitled **“**Outdoor residual insecticide spraying, a new approach for the control of the exophilic vectors of human visceral leishmaniasis: Phlebotomus orientalis in East Africa” describes an interesting study. The results of this study are of major eco-epidemiological importance and should lead to a better control of visceral leishmaniasis in East Africa. The manuscript should be published after taking into consideration the following comments:

**Introduction**

The authors should report the curve showing the phenology of *P. orientalis* in

Soudan.

**Methods**

The authors should use the sticky traps similarly to light traps placed 1 m above the ground and not placed on the ground. ST traps should be used to report the densities of *P. orientalis* (number of sandflies per m² of ST).

The authors should use the Abott’s formula to report the reduction in the abundance of sandflies.

In addition to reduction of sandfly’s abundance, the author should report the infection rate of *P. orientalis* with *L. donovani*.

**Conclusion**

While IRS and ITN showed their limitations, this approach has a great potential to be used in controlling VL in East Africa where the number of cases exceeded the Indian subcontinent.
